# Tracking Metabolite Variations during the Degradation of Vegetables in Rice Bran Bed with Intact-State Nuclear Magnetic Resonance Spectroscopy

**DOI:** 10.3390/metabo14070391

**Published:** 2024-07-19

**Authors:** Kengo Ito, Ryusei Yamamoto, Yasuyo Sekiyama

**Affiliations:** 1 Research Center for Agricultural Information Technology, National Agriculture and Food Research Organization, Tsukuba 305-0856, Japan; 2 Research Center for Advanced Analysis, National Agriculture and Food Research Organization, Tsukuba 305-8642, Japan

**Keywords:** nuclear magnetic resonance, intermolecular single quantum coherence, intact tissue, inhomogeneous magnetic field, vegetable degradation, fermentation, metabolic pathway

## Abstract

Fermentation—a process of compound degradation by microorganisms—is a traditional food processing method utilized worldwide for the long-term preservation of fresh foods. In recent years, fermented foods have gained attention as health foods. Fermentation increases the nutritional value of ingredients, producing complex flavors and aromas. To identify unknown components in fermented foods, it is necessary to analyze compounds and conditions nondestructively and comprehensively. We performed intact-state nuclear magnetic resonance (NMR) spectroscopy using intermolecular single quantum coherence (iSQC) to detect the degradation of vegetables directly and nondestructively. We used two types of vegetables and a rice bran bed (nukazuke), which is used for traditional vegetable fermentation in Japan. Major metabolites such as saccharides, organic acids, and amino acids were identified in iSQC-sliced spectra. Comparing NMR signal intensities during degradation revealed the transition of metabolites characteristic of lactic acid fermentation. A pathway-based network analysis showed pathways involved in amino acid metabolism and lactic acid fermentation. Our analytical approach with intact-state NMR spectroscopy using iSQC demonstrated that it may be effective in other experimental systems, allowing for the evaluation of phenomena that have been conventionally overlooked in their true state.

## 1. Introduction

Food is processed using several methods that alter its taste, flavor, and texture to enhance its nutritional value and provide sensory satisfaction. Fermentation stands out as a microorganism-mediated chemical transformation of food that produces healthy compounds. Recently, fermented foods, such as natto and kimchi, have gained attention for their positive impact on human gastrointestinal health, leading to intensified research and development efforts [1]. Fermentation induces various alterations in the appearance, nutrient composition, taste, flavor, and texture of foods and extends the shelf life of foods. Fermentation has been used for millennia, resulting in the production of a range of fermented foods [2,3,4]. Traditional Japanese fermented foods constitute a vital element of Washoku cuisine, recognized as a UNESCO Intangible Cultural Heritage in 2013. Fugu roe, a traditional Japanese delicacy that undergoes prolonged fermentation on a rice bran bed to eliminate toxic substances, is an intriguing example of these fermented foods [5]. Uncontrolled fermentation can lead to excessive fermentation and changes in the microflora, resulting in undesirable reactions. This is referred to as “spoilage”, which produces an unpleasant odor or harmful compounds, making food unfit for consumption.

The field of foodomics has emerged to assess the ingredients, quality, and safety of these foods. Its origins trace back to the 19th century, when researchers began isolating macroscopic components using simple pH meters and chromatography methods. However, in modern times, sophisticated analytical instruments, such as high-resolution mass spectrometry (MS) and high-resolution nuclear magnetic resonance (NMR), have become prevalent [6]. Gas chromatography–mass spectrometry (GC–MS) and liquid chromatography–mass spectrometry (LC–MS) have become mainstream. These analytical methods involve extraction, derivatization, and chromatographic separation, enabling the evaluation of trace compounds in foods [7]. NMR offers the significant advantage of a straightforward extraction method and the ability to assess compounds without specific targets. Consequently, it is feasible to detect compounds and assess chemical changes during fermentation [8,9]. However, these analytical methods, including NMR, typically necessitate the extraction of target compounds before the subsequent measurements, making it challenging to nondestructively detect and evaluate compounds in food. Moreover, drawbacks exist, such as that the compounds detected depend on the polarity of the extraction solvent and that heavy solvents are required for solution-state NMR.

Therefore, in vivo NMR and magnetic resonance spectroscopy are effective for evaluating compounds in foods nondestructively. However, signal broadening due to an inhomogeneous magnetic field proves to be a challenge in NMR measurements utilizing intact samples, as employed in in vivo NMR and magnetic resonance spectroscopy. In a typical solution-state NMR measurement employing a homogeneous solution sample, an inhomogeneous magnetic field can be corrected by adjusting the shim current, thus yielding high-resolution NMR spectra. Conversely, when employing an intact sample with a significant inhomogeneous magnetic field in an NMR measurement, complete elimination of the inhomogeneous magnetic field with a shim current proves difficult. Consequently, NMR spectra acquired from normal intact-state NMR measurements appear broad, making it challenging to acquire high-resolution NMR spectra that identify metabolites, compared with conventional solution-state NMR that uses homogeneous solution samples [10,11].

This problem can be solved by using high-resolution magic-angle spinning (HR-MAS), which can acquire high-resolution NMR spectra of inhomogeneous samples that yield broad unresolved NMR spectra using conventional solution-state NMR measurement [11]. Numerous in vivo NMR measurements employing intact samples through HR-MAS have been documented, facilitating the identification of metabolite-derived signals without the need for extraction processes [12,13]. However, due to the high-speed rotation of the sample rotor, biological samples are at a risk of tissue damage, thus preventing completely nondestructive measurements. In recent years, alternative techniques have emerged to acquire high-resolution NMR spectra unaffected by inhomogeneous magnetic fields. One such method is the intermolecular single quantum coherence (iSQC) NMR pulse sequence, which enables the acquisition of high-resolution NMR spectra from intact samples without the use of MAS [14,15].

In this study, we evaluated Japanese pickled vegetables in a rice bran bed using intact-state NMR spectroscopy. In Japan, a rice bran bed as a fermentation bed is popularly known as “nukazuke”. Japanese pickled cucumber and carrot are common foods in Japan. Therefore, we chose carrot and cucumber as degradation targets and rice bran as the fermentation bed to track metabolite variations during degradation using intact-state NMR spectroscopy. We used iSQC to track metabolite variations in vegetables during degradation, including fermentation and putrefaction. Additionally, we compared iSQC-sliced spectra and other 1D ^1^H NMR spectra to assess the performance of intact-state NMR spectroscopy. Finally, we performed a metabolic pathway analysis based on the metabolic information provided by NMR measurements. Metabolic pathways related to the degradation process were investigated using a pathway-based network analysis.

## 2. Materials and Methods

### 2.1. Raw Materials

Carrots (*Daucus carota* subsp. *sativa*) and cucumbers (*Cucumis sativus*) were purchased from a local fruit and vegetable store (Tsukuba, Japan) in March and April 2024, respectively. Rice bran was purchased from a local rice retailer (Kawagoe, Japan) in March 2024. The ingredients were stored at 4 °C until use.

### 2.2. Degradation Experiments

A rice bran bed was prepared by mixing rice bran, 48 g of sodium chloride (guaranteed reagent grade, FUJIFILM Wako Pure Chemical Corporation, Osaka, Japan), and 400 mL of Elix water (Elix UV 5, Merck Millipore, Burlington, MA, USA) in a 100 mL glass bottle. Raw carrots and cucumbers were finely chopped into ~0.5 cm pieces. The chopped vegetables and rice bran bed were mixed at a weight ratio of 9:1, placed in a 100 mL glass jar, and incubated in an incubator (LET-2100, EYELA, Tokyo, Japan) at 25 °C in the dark for up to 1 week (Figure S1). A control degradation experiment was set up using the rice bran bed. The pH and water content of the rice bran bed were monitored on days 0, 3, and 7. The pickled vegetables in the rice bran bed were collected on days 3 and 7. The rice bran was rinsed off from the collected vegetables with Milli-Q water (Milli-Q, Advantage, Merck Millipore, Burlington, MA, USA). The vegetables were gently wiped with paper towels. An aliquot of the pickled vegetables was directly utilized for intact-state NMR measurements, while the remaining sample was immediately frozen in liquid nitrogen.

### 2.3. Sample Preparation for NMR Measurements

For intact-state NMR measurements, raw vegetables were hollowed out using a 5 mm diameter cork-borer and inserted into a 5 mm NMR tube (Sigemi Co., Tokyo, Japan). The above aliquot of vegetables was inserted up to a height of approximately 5 cm into the 5 mm NMR tube (Figure S1).

For solution-state NMR measurements, the frozen samples were freeze-dried with a lyophilizer at 25 °C (FD-20BU/SK03, Nihon Techno Service, Ibaraki, Japan) and ground into a fine powder. Metabolites were extracted using a D_2_O-based potassium phosphate buffer as described in [16]. The extract was placed in a 5 mm NMR tube. Rice bran collected on days 0, 3, and 7 in the control degradation experiment using only rice bran was prepared under the same conditions.

### 2.4. NMR Measurements

All intact-state NMR spectra were acquired on a Bruker Avance III 600 NMR spectrometer (Bruker BioSpin GmbH, Rheinstetten, Germany) equipped with a 5 mm TXI probe at 298 K, and all intact-state NMR measurements were processed using TopSpin 3.5 software (Bruker BioSpin GmbH, Rheinstetten, Germany). B_0_ shimming was performed using a homogeneous solution-state sample before all intact-state NMR measurements. A pulse length of 90°, transmitter frequency offset, and tuning and matching of the NMR probe were optimized for each intact sample. Conventional 1D ^1^H NMR spectra were acquired using the Bruker standard pulse program “zg”. The acquisition parameters were as follows: time domain size of 32,768, sweep width in acquisition direction of 16 ppm, scan number of 32, and relaxation delay of 3.5 s. WATERGATE spectra were acquired using the Bruker standard pulse program “zggpw5”. The acquisition parameters were as follows: time domain size of 32,768, sweep width in acquisition direction of 16 ppm, scan number of 32, relaxation delay of 3.5 s, delay for binomial water suppression of 104 μs, W5 first gradient pulse of 18 G/cm, and W5 second gradient pulse of 11.7 G/cm. An intermolecular dipolar-interaction enhanced all lines (IDEAL-III) pulse sequence with radiation damping compensation, delays adjusted such that the sequence forms a spin-echo, and W5 WATERGATE as water suppression [17] were used as iSQC. The acquisition parameters were as follows: time domain size of 8192 (F2) and 96 (F1), sweep width in acquisition direction of 12 ppm (F2) and 600 Hz (F1), sweep, scan number of 192, relaxation delay of 3.5 s, water selective pulse length of 5 ms, iSQC first gradient pulse of −5.3 G/cm, iSQC second gradient pulse of −3.7 G/cm, iSQC third gradient pulse of 12.7 G/cm, iSQC echo time of 15–40 ms, delay for binomial water suppression of 139 μs, W5 first gradient pulse of 14.8 G/cm, and W5 second gradient pulse of 26 G/cm.

All solution-state NMR spectra were acquired on a Bruker Avance NEO 800 NMR spectrometer (Bruker BioSpin GmbH, Rheinstetten, Germany) equipped with a SampleJet automatic sample changer and a 5 mm Cryo TCI probe at 298 K. All solution-state NMR measurements were performed using the IconNMR 6.1 software included in TopSpin 4.3. Water-presaturated 1D ^1^H NMR spectra were acquired using the Bruker standard pulse program “zgpr”. The acquisition parameters were as follows: time domain size of 65,536, sweep width in acquisition direction of 20 ppm, scan number of 128, and relaxation delay of 4 s.

### 2.5. Data Processing and Analysis

NMR data were preprocessed using TopSpin 4.1.1 software. For the conventional 1D ^1^H NMR spectra and WATERGATE spectra of intact samples, the size of the real spectrum was 32,768 and the line broadening for the exponential multiplication (EM) window function was 0.3 Hz. These spectral phases were corrected manually. For the iSQC spectra of intact samples, the size of the real spectrum was 16,384 (F2) and 128 (F1) and the line broadening was 0.1 Hz with the EM window function (F2) and sine window function with a 0-shifted sine-bell. These iSQC spectra were Fourier transformed in magnitude mode. Then, 1D iSQC spectra were collected by slicing around the center on the F1 axis of the 2D iSQC spectra. All intact-state NMR spectra were calibrated by referring to the solution-state NMR spectra. For the water-presaturated 1D ^1^H NMR spectra of extracts from vegetables and the rice bran bed, the size of the real spectrum was 65,536 and the line broadening for the EM window function was 0 Hz. These spectral phases were corrected manually.

After preprocessing NMR data on TopSpin 4.1.1 software, nmrglue 0.10 in python [18] was used for converting the NMR binary data to a numeric matrix and performing baseline correction, and pynmranalysis 1.1.3 in python (https://github.com/1feres1/pynmranalysis/, accessed on 13 June 2024) was used for binning and normalization. Binning was performed using a 0.002 ppm bin size with the composite Simpson’s rule for integration. NMR spectra were normalized using a mean normalization method. Solution-state NMR spectra were normalized using a peak normalization method with a sodium trimethylsilylpropanesulfonate (DSS) peak.

The metabolite annotations and the collection of its concentrations to each peak of the solution-state NMR spectra were performed by semi-automated peak fitting using Chenomx NMR Suite 10.1 software including a 500 MHz ^1^H-NMR library [19]. Metabolite annotation for each peak of the iSQC spectra was performed with reference to the metabolite annotations of the solution-state NMR spectra.

A metabolic pathway analysis was performed using MetaboAnalyst 6.0 based on the Kyoto Encyclopedia of Genes and Genomes (KEGG) database [20]. The KEGG pathway library of lactobacillus in prokaryotes was selected as the analytical parameter for pathway analysis [21]. The pathway-based network analysis of the results was performed using Cytoscape 3.8 with the KEGGscape 0.9.1 plugin [22,23].

## 3. Results

### 3.1. Performance of iSQC Experiment for Intact Samples

First, we sought to identify the most effective intermolecular multiple quantum coherence pulse sequences for the intact-state NMR spectroscopy to assess the quality of the raw and pickled vegetables. The performance of the pulse sequences was evaluated under an artificially generated inhomogeneous magnetic field by deshimming and using standard solutions comprising glucose, fructose, sucrose, citrate, malate, acetate, and ethanol in D_2_O. We verified pulse sequences for constant time correlation spectroscopy (CT-COSY) [24], IDEAL-I [25], IDEAL-II [26], IDEAL-III [27], and in-phase iSQC [17]. For CT-COSY, high-resolution NMR spectra could be acquired by dispersing broad NMR signals along the F1 axis without any problem with signal detection sensitivity. However, generating a 1D NMR spectrum was difficult due to the overlapping of cross-peaks. Cross-peaks were suppressed in IDEAL-I compared with CT-COSY, making NMR signal identification easier. Nonetheless, there was a problem with the *J*-splitting being subject to scaling. For IDEAL-II, NMR signals could be detected at the center of the F1 axis, making the generation of 1D NMR spectra easier than that with COSY and IDEAL-I. However, there was a problem with the *J*-splitting being subject to scaling. For IDEAL-III, NMR signals were detected at the center of the F1 axis, facilitating 1D NMR spectrum generation, and spectra comparable to those under a homogeneous magnetic field were achieved. NMR signal detection sensitivity was better with in-phase iSQC than with IDEAL-III. This was due to the suppression of radiation damping during the evolution period using the *z*-axis gradient. However, there was no significant improvement in NMR signal detection sensitivity with the adiabatic spin lock pulse, and the fine-tuning of echo time was challenging. Therefore, we chose IDEAL-III with radiation damping compensation as the iSQC pulse sequence for this study.

We adjusted the acquisition parameters for iSQC measurement using intact, cylindrically cut raw carrots and cucumbers (Figures S2a and S3a). Unlike simple solution-state samples, intact vegetables contain metabolites localized in organelles and tissues. The presence of various macromolecules increases intracellular viscosity in intact vegetables. Therefore, the mobility of each metabolite is restricted, resulting in shorter magnetic relaxation times compared with solution-state samples. Because the degree of the inhomogeneous magnetic field was unknown, we had to adjust the acquisition parameters initially. An acquisition time of approximately 150 ms in the F1 axis as the evolution direction was optimal, because signals were barely detected beyond this duration. Consequently, the free induction decay (FID) resolution was ~6 Hz in the F1 axis as the evolution direction. The full width at half maximum of each peak was 30–50 Hz in the F1 axis as the evolution direction. An echo time of 15–40 ms in the iSQC pulse sequence was found to be optimal, because signal detection sensitivity decreased with longer echo times. The optimal first gradient pulse strength for iSQC was −5.3 G/cm, because higher strengths led to decreased NME signal detection sensitivity. To enhance NMR signal detection, the number of scans was set to 192, resulting in an experimental time of ~18 h.

For carrots and cucumbers on days 3 and 7 of degradation, iSQC measurements were performed using the same acquisition parameters (Figures S2b,c and S3b,c). NMR signals of saccharides, organic acids, and amino acids were prominent. Although the NMR signals of saccharides were prominent on day 0 of degradation, the NMR signal intensity decreased as degradation progressed. Several NMR signals of organic acids and amino acids showed an increasing trend in intensity with the progress of degradation.

**Figure 1:**
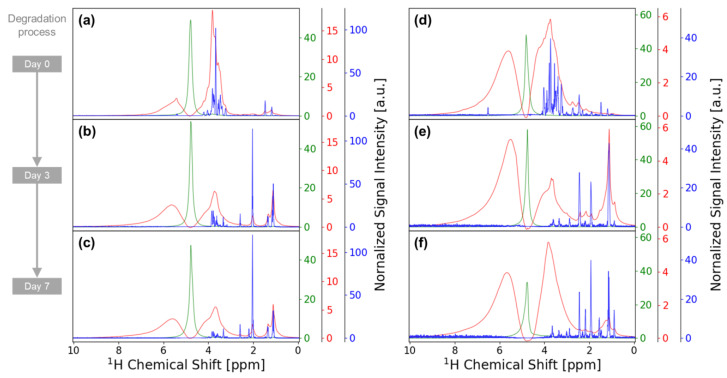
The 1D ^1^H NMR spectra of (**a**) intact raw carrot and Japanese pickled carrot on (**b**) day 3 and (**c**) day 7. The 1D ^1^H NMR spectra of (**d**) intact raw cucumber and Japanese pickled cucumber on (**e**) day 3 and (**f**) day 7. Conventional 1D ^1^H NMR spectra (green), WATERGATE spectra (red), and iSQC-sliced spectra (blue) are overlayed on the same box for each degradation process.

iSQC-sliced spectra were acquired from 2D iSQC spectra for evaluating the performance of spectral resolution compared with conventional NMR measurements (Figure 1). The NMR signal from water was very strong in the conventional 1D ^1^H NMR spectra because vegetables contain a high amount of water, and NMR signals from other metabolites were hardly detected. Therefore, 1D ^1^H NMR spectra suppressed with water signal were acquired using the WATERGATE method. In the WATERGATE spectra, although each peak width was broad, the presence of saccharide signals could be confirmed on day 0 of degradation (Figure 1a,d), and amino acid or organic acid signals could be confirmed on days 3 and 7 of degradation (Figure 1b,c,e,f). As for the spectral performance, the full width at half maximum of a strong peak at ~2 ppm in the carrot spectrum on day 3 of degradation was approximately 37 Hz with the WATERGATE spectra, whereas it was ~11 Hz with the iSQC-sliced spectra, resulting in approximately three times sharper peaks (Figure 1b). Additionally, the saccharide signals at 3–4 ppm for raw cucumber appeared as a single large peak in the WATERGATE spectra but could be resolved into >10 peaks in the iSQC-sliced spectra (Figure 1d).

### 3.2. Metabolite Variation during Vegetable Degradation

**Figure 2:**
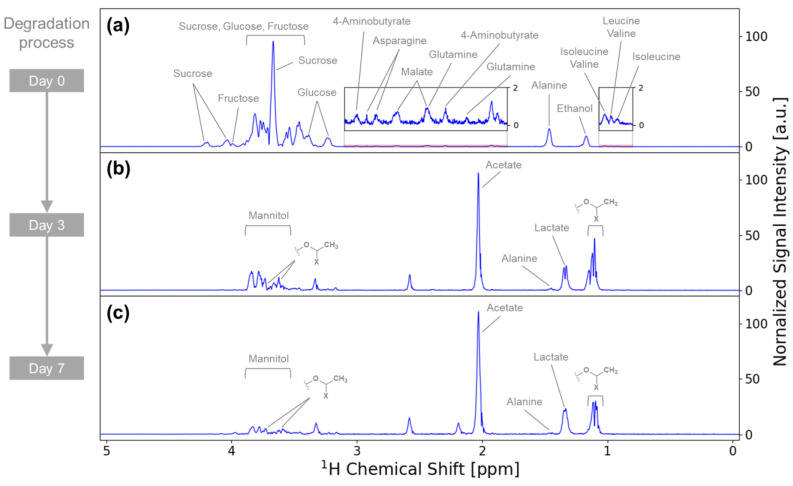
The 1D iSQC-sliced spectra of (**a**) intact raw carrot and Japanese pickled carrot on (**b**) day 3 and (**c**) day 7. Each signal is annotated with the metabolites and the partial structure of the unknown metabolite. “X” in the partial structure of the unknown metabolite is expected to be a heteroatom, although its identity remains to be confirmed.

Metabolite annotation was performed on the detected NMR signals before tracking the variations in metabolites during the degradation of vegetables. To annotate the metabolites to each signal in the iSQC spectra, we also performed solution-state NMR measurements of its extracts. The NMR spectra of carrot extracts resulted in the annotation of 42 metabolites, including saccharides, organic acids, amino acids, and aromatic compounds (Figure S4). Similarly, the NMR spectra of cucumber extracts led to the annotation of 45 metabolites, including saccharides, organic acids, amino acids, and aromatic compounds (Figure S5). Referring to the metabolite annotation from these NMR spectra of extracts, we annotated the metabolites to each peak of the iSQC spectra. The iSQC spectra of the carrots resulted in the annotation of 15 metabolites, including saccharides, organic acids, and amino acids (Figure 2a–c). Furthermore, we performed a partial structure annotation of the unknown metabolite for signals prominently detected on days 3 and 7 of carrot degradation (Figure 2b,c). The unidentified major NMR signals from the partial structure of the unknown metabolite at 1.1, 3.6, and 3.7 ppm were estimated using various 2D NMR techniques, such as ^1^H–^1^H total correlation spectroscopy, ^1^H–^13^C heteronuclear single quantum coherence, and ^1^H–^13^C heteronuclear multiple bond coherence, on solution-state NMR.

**Figure 3:**
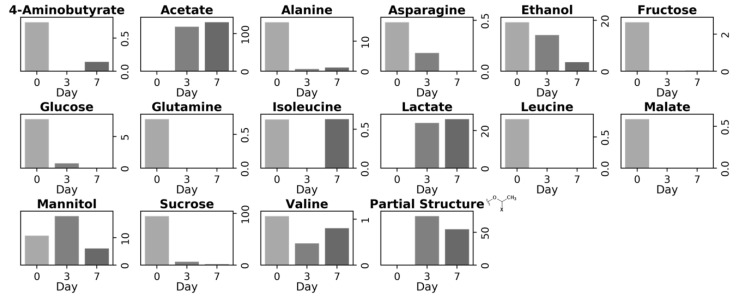
Transition of iSQC signal intensities of each metabolite and the methyl group of the unknown metabolite in carrots on each day of degradation. The iSQC signal intensities were collected from the normalized 1D iSQC-sliced spectra shown in Figure 2.

The variations in the metabolites of the carrots during degradation were examined in detail using the normalized NMR signal intensities of the iSQC spectra (Figure 3). The signal intensities of 4-aminobutyrate, alanine, asparagine, ethanol, fructose, glucose, glutamine, leucine, malate, and sucrose were prominent on day 0 but showed a decreasing trend or were scarcely detected after day 3. By contrast, the signal intensities of acetate, lactate, and the partial structure of the unknown metabolite were prominent on days 3 and 7 of degradation but hardly detected on day 0 of degradation. These variations in the metabolites of the carrots were confirmed by comparing the concentration of each metabolite detected in solution-state NMR (Figure S6). Alanine, asparagine, ethanol, fructose, glucose, glutamine, malate, and sucrose were detected by both solution-state NMR and iSQC measurements. Their signal intensities were prominent on day 0 of degradation but scarcely detected after day 3. Choline, formate, glutamate, glycine, and isoleucine were detected only by solution-state NMR. Their signal intensities were prominent on day 0 of degradation and showed a decreasing trend or were scarcely detected after day 3 of degradation. Acetate and lactate were detected by both solution-state NMR and iSQC. Both metabolites were prominently detected on days 3 and 7 of degradation but were hardly detected on day 0 of degradation. 2-Aminobutyrate, 2-hydroxyisovalerate, 3-hydroxy-3-methylglutarate, citrate, formate, and others were detected prominently only by solution-state NMR on days 3 and 7 of degradation.

**Figure 4:**
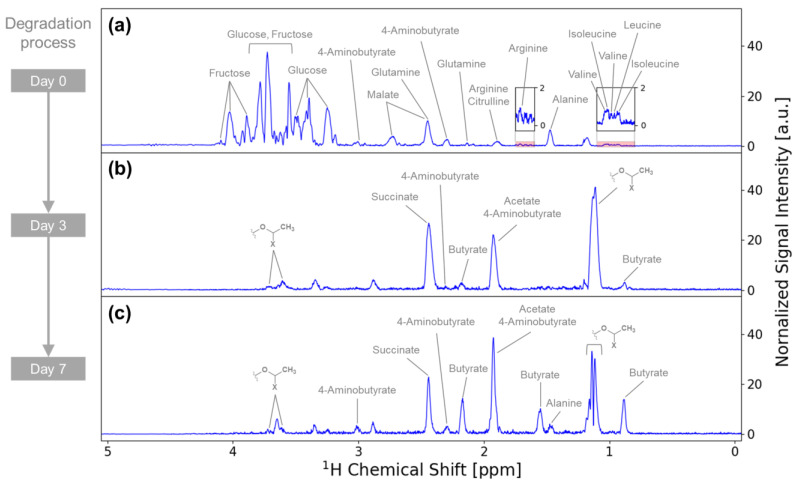
The 1D iSQC-sliced spectra of (**a**) intact raw cucumber and Japanese pickled cucumber on (**b**) day 3 and (**c**) day 7. The metabolites and the partial structure of the unknown metabolite are annotated on each signal. Label “X” in the partial structure of the unknown metabolite is expected to be a heteroatom, although its identity remains to be confirmed.

**Figure 5:**
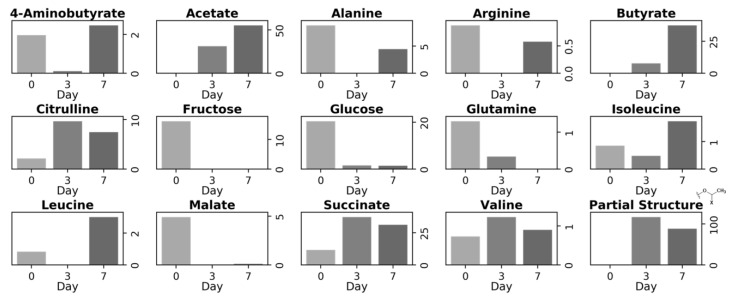
Transition of iSQC signal intensities of each metabolite and the methyl group of the unknown metabolite in cucumber on each day of degradation. The iSQC signal intensities were collected from the normalized 1D iSQC-sliced spectra shown in Figure 4.

Metabolite annotation of the iSQC spectra of cucumber annotated 14 metabolites, including saccharides, organic acids, and amino acids (Figure 4a–c). In addition, the NMR signals from the partial structure of the unknown metabolite that were prominent on days 3 and 7 of degradation were annotated (Figure 4b,c). The variations in metabolites in the cucumbers during degradation were examined using the normalized signal intensities of the iSQC spectra (Figure 5). Fructose, glucose, glutamine, and malate were prominently detected on day 0 of degradation but were scarcely detected after day 3 of degradation. By contrast, acetate, butyrate, succinate, and the unknown metabolite were prominently detected on days 3 and 7 but were hardly detected on day 0 of degradation. These variations in metabolites in the cucumbers were confirmed by comparing the concentration of each metabolite detected in solution-state NMR (Figure S7). Fructose, glucose, glutamine, and malate were detected using both solution-state NMR and iSQC measurements, with prominent signals on day 0 of degradation and negligible signals after day 3 of degradation. Asparagine, aspartate, choline, citrate, and fumarate were detected by only solution-state NMR measurement, with prominent signals on day 0 of degradation and a decreasing trend or negligible signals after day 3 of degradation. Acetate, butyrate, and succinate were detected by both solution-state NMR and iSQC, mainly on days 3 and 7 of degradation, but were hardly detected on day 0 of degradation. 2-Aminobutyrate, 2-hydroxyisovalerate, 3-hydroxy-3-methylglutarate, formate, isovalerate, etc., were detected only by solution-state NMR, mainly on days 3 and 7 of degradation.

Solution-state NMR measurements of extracts from the rice bran bed used in the control experiment annotated 89 metabolites, including saccharides, organic acids, amino acids, and aromatic compounds (Figure S8). Of these, 58 were unique to rice bran bed, whereas 31 were present in raw and/or Japanese pickled vegetables. The variations in these metabolites in rice bran bed were examined (Figure S9). Sucrose was prominently detected on day 0 of degradation and showed a decreasing trend after day 3 of degradation. Acetate and lactate were prominently detected on days 3 and 7 of degradation but were hardly detected on day 0 of degradation. These metabolites were consumed and produced even when vegetables were not included with rice bran bed.

### 3.3. Investigation of the Metabolic Pathways Related to Degradation

**Figure 6:**
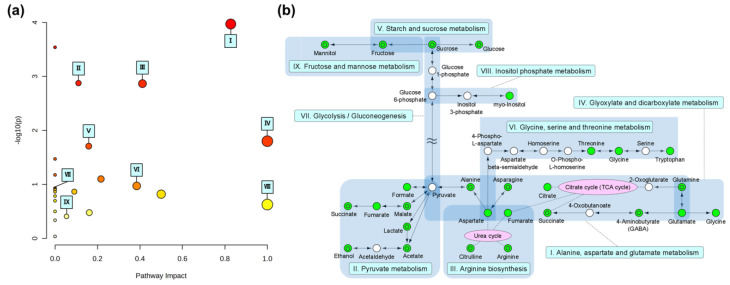
(**a**) Summary of metabolic pathway analysis using metabolites detected through NMR measurements. The pathways are plotted as nodes according to *p*-values as significance on the *y*-axis and pathway impact values on the *x*-axis. Node color is based on *p*-value and node radius is determined based on pathway impact value. Most contributing pathways are in the top right corner. Each node represents the following metabolic and biosynthetic pathways: I—alanine, aspartate, and glutamate metabolism; II—pyruvate metabolism; III—arginine biosynthesis; IV—glyoxylate and dicarboxylate metabolism; V—starch and sucrose metabolism; VI—glycine, serine, and threonine metabolism; VII—glycolysis or gluconeogenesis; VIII—inositol phosphate metabolism; and IX—fructose and mannose metabolism. (**b**) Pathway-based network constructed from the results of the pathway analysis. The green nodes indicate metabolites detected through NMR spectra; double-circled nodes indicate metabolites detected through iSQC-sliced spectra. Blue blocks represent metabolic and biosynthetic pathways, and nodes related to these are grouped together. Arrows connecting nodes indicate the direction of metabolism or biosynthesis. Purple nodes represent the urea cycle and the TCA cycle, and related nodes are connected by dotted lines.

Metabolic and biosynthetic pathways involved in the degradation of vegetables in rice bran were investigated based on the metabolites annotated in this study. A pathway analysis was performed using a library of metabolic and biosynthetic pathways of lactobacillus associated with vegetable degradation in rice bran (Figure 6a). The results confirmed the relevance of 24 metabolic and biosynthetic pathways. The pathways for alanine, aspartate, and glutamate metabolism had the highest −log_10_(*p*) values and significant impact values, indicating their importance. Additionally, pyruvate metabolism and arginine biosynthesis had high −log_10_(*p*) values, indicating that they were highly important.

A pathway-based network analysis was performed to investigate the relationships between the major metabolic and biosynthetic pathways identified from the pathway analysis (Figure 6b). First, the relationships between alanine, aspartate, and glutamate metabolism; pyruvate metabolism; and arginine biosynthesis were examined. Fumarate and aspartate, which are involved in alanine, aspartate, and glutamate metabolism, correlated with the urea cycle in arginine biosynthesis. These metabolic pathways have been well established in plant fermentation processes [28,29]. Pyruvate, which is involved in alanine, aspartate, and glutamate metabolism, correlated with pyruvate metabolism. Pyruvate was connected through the long pathway in glycolysis and serves as a hub for alanine, aspartate, and glutamate metabolism.

Glycolysis was expected to correlate with starch and sucrose metabolism and fructose and mannose metabolism. Glucose 6-phosphate from glycolysis was expected to correlate with inositol phosphate metabolism, which had a high impact value. This metabolism was reported to be encoded by a gene from *Lactiplantibacillus plantarum*, often isolated from fermented vegetables [30,31]. Glyoxylate and dicarboxylate metabolism, which had a high impact (impact value = 1), correlated with alanine, aspartate, and glutamate metabolism. Although the importance of glycine, serine, and threonine metabolism was not high, they were expected to correlate with the urea cycle in arginine biosynthesis. Lactic acid fermentation of vegetables was related to starch and sucrose metabolism; glycine, serine, and threonine metabolism; and alanine, aspartate, and glutamate metabolism [32]. Other metabolic and biosynthetic pathways were not considered highly significant in the pathway analysis, and connecting them to these metabolic pathways was challenging.

## 4. Discussion

Similar to other studies that used intermolecular multiple quantum coherence measurements with intact insect and fruit samples [17,33], we acquired well-resolved NMR spectra of intact raw and pickled Japanese vegetables. These spectra were better than conventional NMR spectra. iSQC was able to extract chemical shift information free of perturbations and provide more detailed chemical structure information for metabolites. Also, iSQC offers an advantage over solution-state NMR spectroscopy in that sample preparation is simple because metabolites need not be extracted. In addition, it can detect metabolites nonpolar to the NMR solvent that are often not observed simultaneously with polar metabolites in solution-state NMR spectroscopy. However, the drawbacks of iSQC are (1) longer experimental times than 1D NMR measurements due to 2D NMR measurements and (2) issues with low signal detection sensitivity due to the short magnetic relaxation times of metabolites in intact samples. Therefore, to detect NMR signals equivalent to those of conventional NMR measurement using iSQC measurement, at least six times the number of scans was required. In addition, limitations in spectral resolution settings makes discerning *J*-couplings with enough resolution challenging with iSQC. Advances combining iSQC with high-speed NMR measurement techniques, such as Ultrafast NMR [34], and high-resolution NMR measurement techniques, such as pure shift NMR [33], are expected to make iSQC more valuable.

The acquired iSQC-sliced spectra had sufficient spectral resolution to identify the NMR peaks of metabolites. Therefore, we could annotate approximately 15 major metabolites and the partial structure of an unknown metabolite on iSQC-sliced spectra by referring to the metabolite annotation from solution-state NMR spectroscopy. Notably, because the intact sample did not undergo pH adjustment, it did not exactly match the NMR peaks of each metabolite in the solution-state NMR. Additionally, some NMR peaks could not be annotated, because they were believed to originate from nonpolar metabolites in the NMR solvent that were not detected by the solution-state NMR. In the future, it is expected that combining iSQC with multi-dimensional NMR measurements, such as ^1^H–^13^C heteronuclear single quantum coherence, ^1^H–^1^H total correlation spectroscopy, and ^1^H–^13^C heteronuclear multiple bond coherence, and/or advanced annotation techniques, including chemical shift prediction by quantum chemical computation and machine learning models [35], will enable a more detailed annotation of metabolites.

Next, we evaluated the transitions of annotated metabolites on the iSQC spectra by assessing NMR signal intensities. Saccharides, such as sucrose, glucose, and fructose, which are abundant in vegetables, showed almost no signals after day 3 of degradation. This was likely due to their consumption and degradation by lactobacillus present in the rice bran [36]. By contrast, organic acids, such as lactate and acetate, showed a gradual increase in signal after day 3 of degradation. These were considered metabolites of the lactobacillus in the rice bran [36,37]. The transition patterns of these metabolites were largely similar with the results from solution-state NMR spectra. It is challenging to perform quantitative evaluations within a single NMR spectrum due to variations in signal intensity caused by different acquisition parameters in iSQC. However, it was considered possible to perform qualitative evaluations between different iSQC spectra with the same acquisition parameters.

Finally, a pathway analysis and pathway-based network analysis were performed to investigate the metabolic and biosynthetic pathways involved in the degradation of vegetables. Given that lactobacillus is predominant in the microbiota of rice bran [38], the pathway analysis was performed by referencing to the lactobacillus library. We characterized the metabolic and biosynthetic pathways of lactobacillus that are associated with the metabolites detected in this study. The pathway-based network analysis enabled the characterization of the relationships between these metabolic pathways. For the major metabolites in the central metabolic pathways of the pathway-based network, most metabolites could be detected even in the iSQC-sliced spectra. The reason for the inability to annotate NMR signals of intermediates with phosphate groups in glycolysis or gluconeogenesis, such as sugar phosphate, was thought to be due to the overlap of these NMR signals with NMR signals of other saccharides, making discrimination difficult. It was believed that these could be characterized by utilizing ^31^P NMR, thus elucidating changes in the chemical form of phosphorus in the rice bran during degradation [39]. Pyruvate was the hub of the three metabolic pathways, and other metabolites could not be annotated through NMR spectroscopy because of their low original concentrations or rapid metabolic rates, leading to their accumulation in trace amounts, making detection impossible. In summary, the analysis of the pathway-based network revealed pathways from saccharides associated with lactic acid fermentation to pyruvate in glycolysis and from pyruvate to lactate [40,41,42]. Amino acid metabolism, which is believed to be associated with lactobacillus, was also identified [43].

## 5. Conclusions

In this study, we evaluated the transition of metabolites during the degradation of vegetables in rice bran as an example of metabolomics research using iSQC. We used iSQC to acquire well-resolved NMR spectra without sample destruction and identified metabolites even in such complex intact samples. The transition of metabolites observed from signal intensities exhibited similar patterns to those from the analytical results using solution-state NMR, enabling qualitative assessment between iSQC-sliced spectra. The metabolic pathway analysis based on obtained metabolite data allowed for the evaluation of major metabolic and biosynthetic pathways, such as lactic acid fermentation and amino acid metabolism, associated with lactobacillus. The analytical approach with intact-state NMR spectroscopy using iSQC demonstrated in this study can be effective in various experimental systems, offering the potential to evaluate phenomena that have been conventionally overlooked in their true state.

## Data Availability

The data presented in this study are available on request from the corresponding author.

## Supplementary Materials

The following supporting information can be downloaded at https://www.mdpi.com/article/10.3390/metabo14070391/s1, Figure S1: Sample preparation scheme for intact-state NMR measurements; Figure S2: Two-dimensional iSQC spectra of intact carrot; Figure S3: Two-dimensional iSQC spectra of intact cucumber; Figure S4: Water-presaturated 1D ^1^H NMR spectra of carrot extracts; Figure S5: Water-presaturated 1D ^1^H NMR spectra of cucumber extracts; Figure S6: Transition of water content and pH in rice bran bed and amount of each metabolite in carrot on each day of degradation; Figure S7: Transition of water content and pH in rice bran bed and amount of each metabolite in cucumber on each day of degradation; Figure S8: Water-presaturated 1D ^1^H NMR spectra of extracts from rice bran bed; Figure S9: Transition of water content and pH in rice bran and amount of each metabolite in rice bran in controlled experiment.
