# Supplementary material for: Tracking Metabolite Variations during the Degradation of Vegetables in Rice Bran Bed with Intact-State Nuclear Magnetic Resonance Spectroscopy
Source: Metabolites. 2024 Jul 19;14(7):391. doi: 10.3390/metabo14070391 (PMC11279010; doi:10.3390/metabo14070391)
Supplement: Supplementary file 1 [file metabolites-14-00391-s001.zip › metabolites-3102276-supplementary.pdf]

Supporting Information

Tracking Metabolite Variations during the Degradation of Vegetables in Rice Bran Bed with Intact-State Nuclear Magnetic Resonance Spectroscopy

Kengo Ito [1,*], Ryusei Yamamoto [2] and Yasuyo Sekiyama [2]

[1] Research Center for Agricultural Information Technology, National Agriculture and Food Research Organization, Tsukuba 305-0856, Japan

[2] Research Center for Advanced Analysis, National Agriculture and Food Research Organization, Tsukuba 305-8642, Japan

* Correspondence: itok428@affrc.go.jp (K.I.)

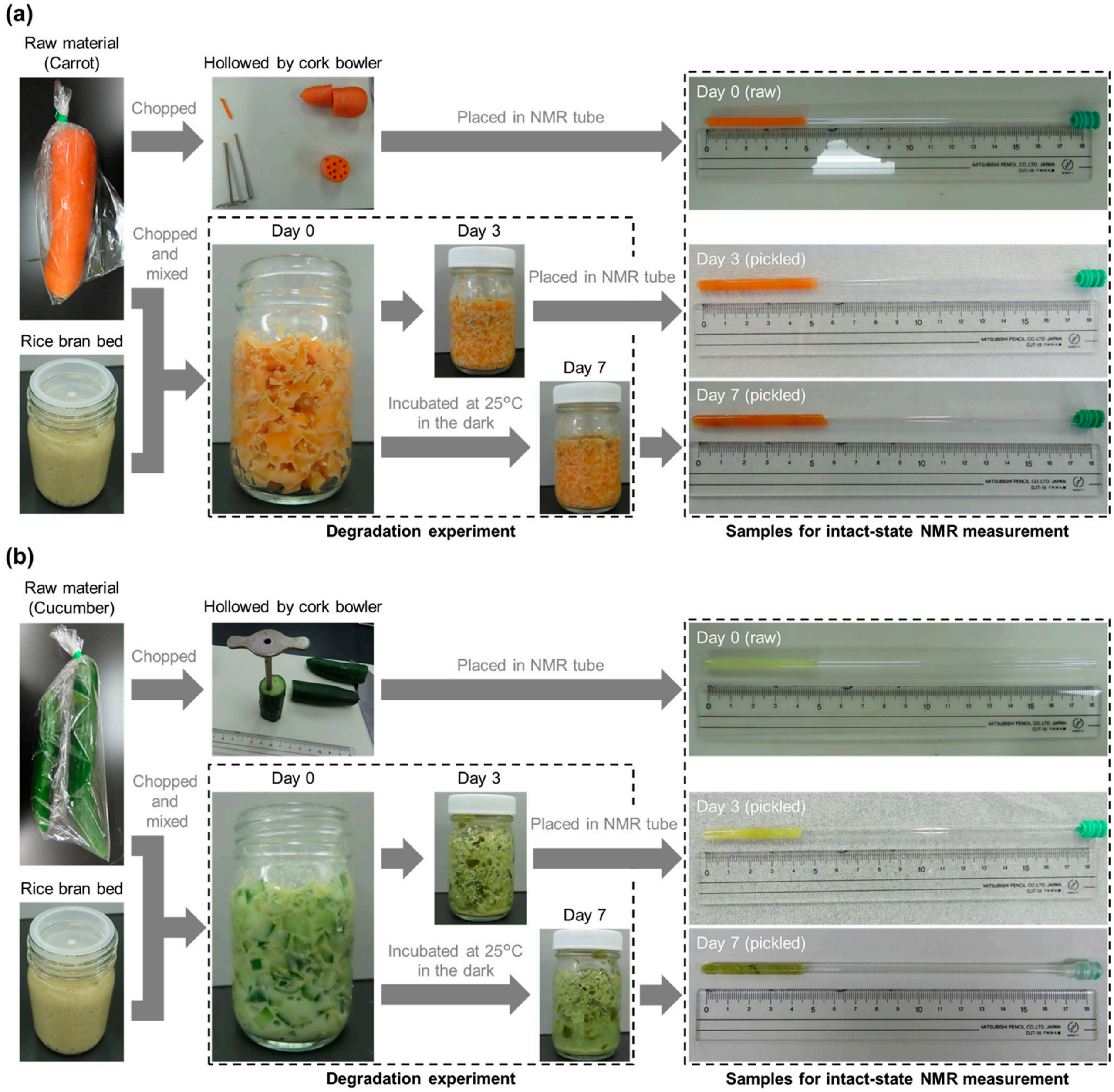

Figure S1. Scheme showing the preparation of raw and Japanese pickled (**a**) carrot and (**b**) cucumber for intact-state nuclear magnetic resonance (NMR) measurement.

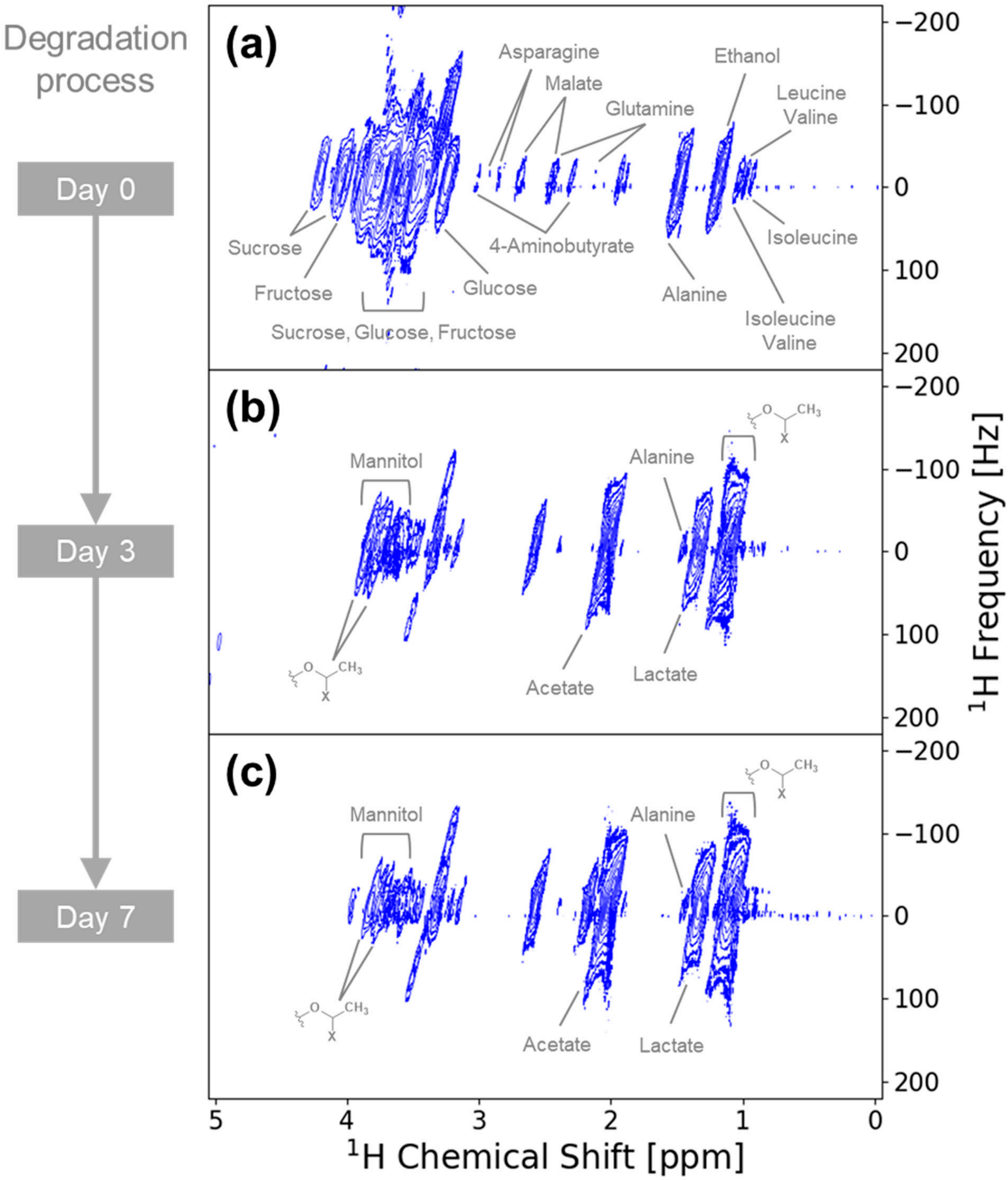

Figure S2. The 2D intermolecular single quantum coherence (iSQC) spectra of (**a**) intact raw carrot, Japanese pickled carrot in rice bran bed on (**b**) day 3 and (**c**) day 7. The metabolites and the partial structure of the unknown metabolite are annotated on each NMR signal. The 1D iSQC-sliced spectra are shown in Figure 2.

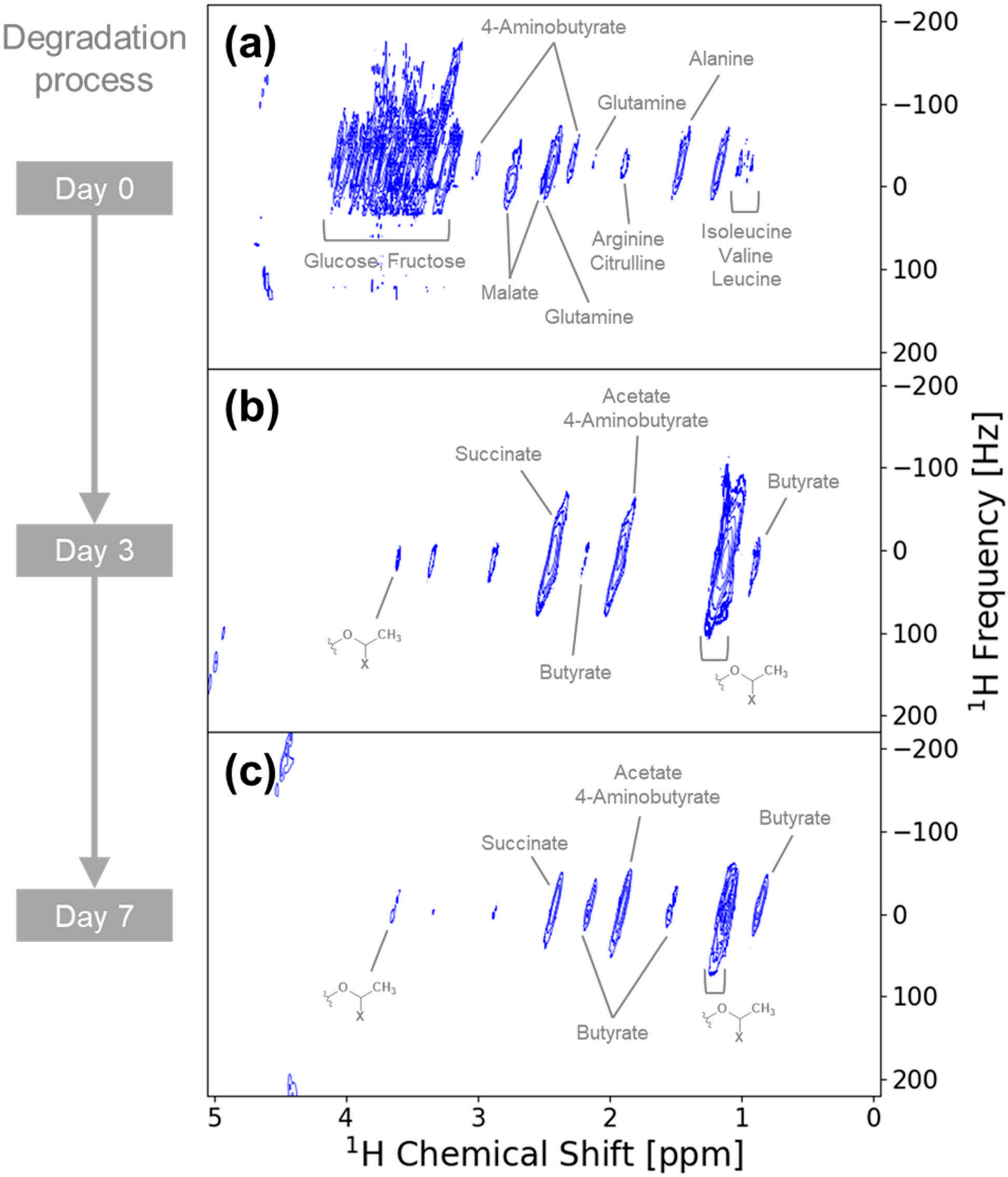

Figure S3. The 2D iSQC spectra of (**a**) intact raw cucumber, Japanese pickled cucumber in rice bran bed on (**b**) day 3 and (**c**) day 7. The metabolites and the partial structure of the unknown metabolite are annotated on each NMR signal. The 1D iSQC-sliced spectra are shown in Figure 4.

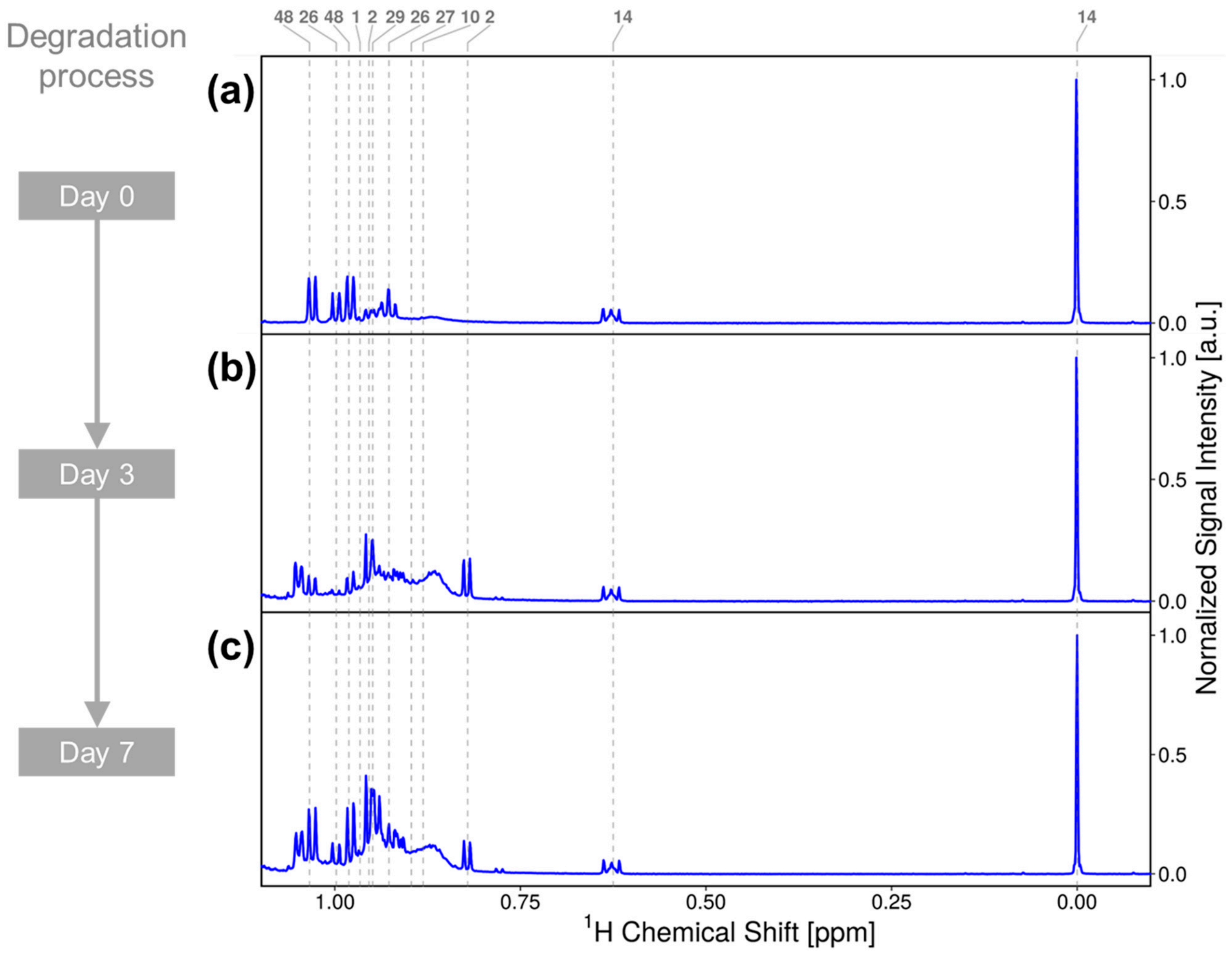

Figure S4. Water presaturated 1D ¹H NMR spectra of extracts from (**a**) raw carrot, Japanese pickled carrot in rice bran bed on (**b**) day 3 and (**c**) day 7. The values shown at the top of the NMR spectra denote the number of annotated metabolites (1: 2-aminobutyrate; 2: 2-hydroxyisovalerate; 3: 3-hydroxy-3-methylglutarate; 4: 4-aminobutyrate; 5: acetate; 6: alanine; 7: arginine; 8: asparagine; 9: aspartate; 10: butyrate; 11: choline; 12: citrate; 13: citrulline; 14: sodium trimethylsilylpropanesulfonate (DSS); 15: ethanol; 16: ethanolamine; 17: formate; 18: fructose; 19: fumarate; 20: glucose; 21: glutamate; 22: glutamine; 23: glycine; 24: histamine; 25: histidine; 26: isoleucine; 27: isovalerate; 28: lactate; 29: leucine; 30: malate; 31: malonate; 32: mannitol; 33: methanol; 34: methionine; 35: nicotinate; 36: phenylalanine; 37: pyrocatechol; 38: pyroglutamate; 39: quinic acid; 40: succinate; 41: sucrose; 42: threonine; 43: trigonelline; 44: tryptophan; 45: tyramine; 46: tyrosine; 47: uridine; 48: valine; and 49: myo-inositol). The dotted lines on the NMR spectra denote chemical shifts of each metabolite registered in Biological Magnetic Resonance Bank (BMRB) (https://bmrb.io/).

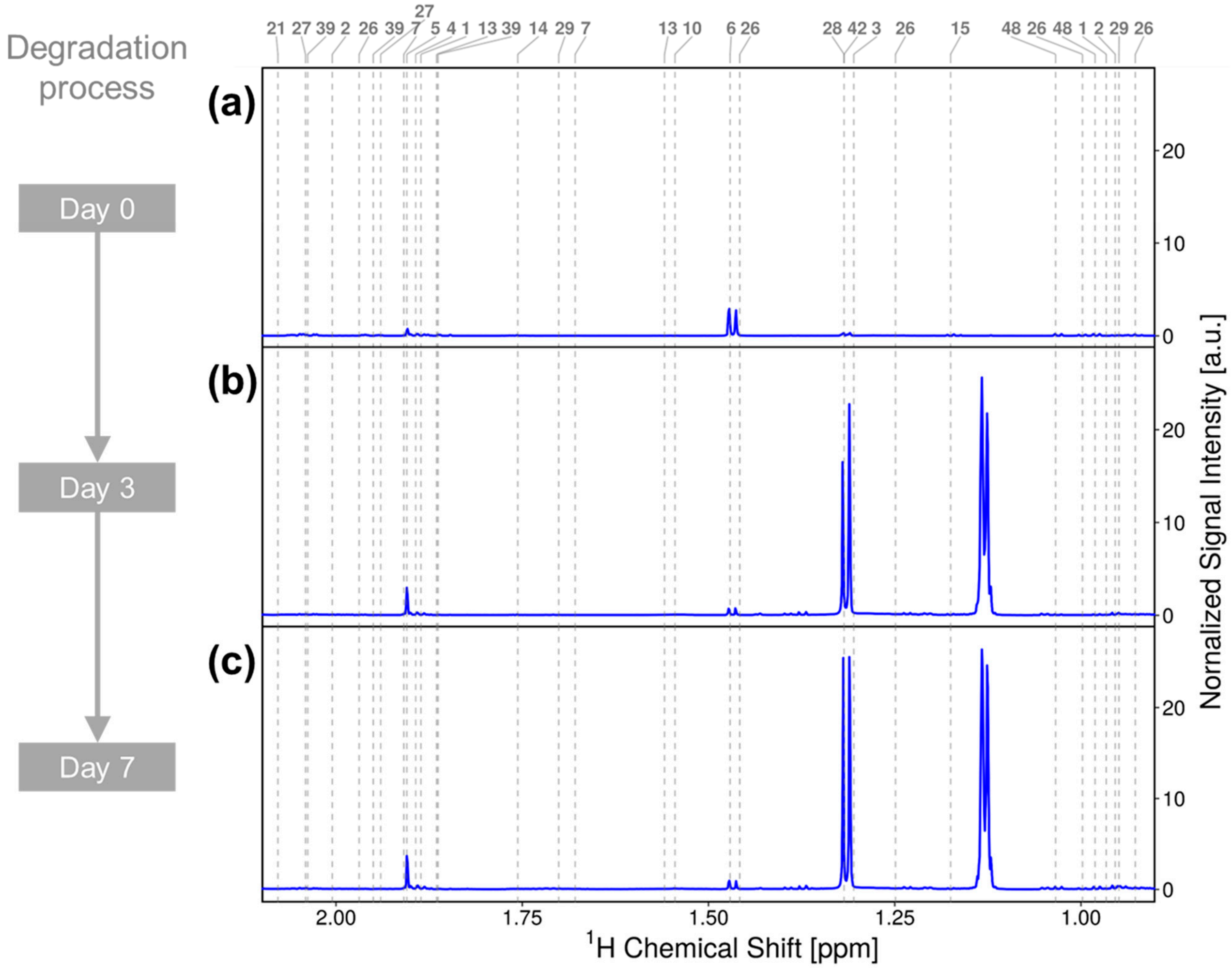

Figure S4. Continued.

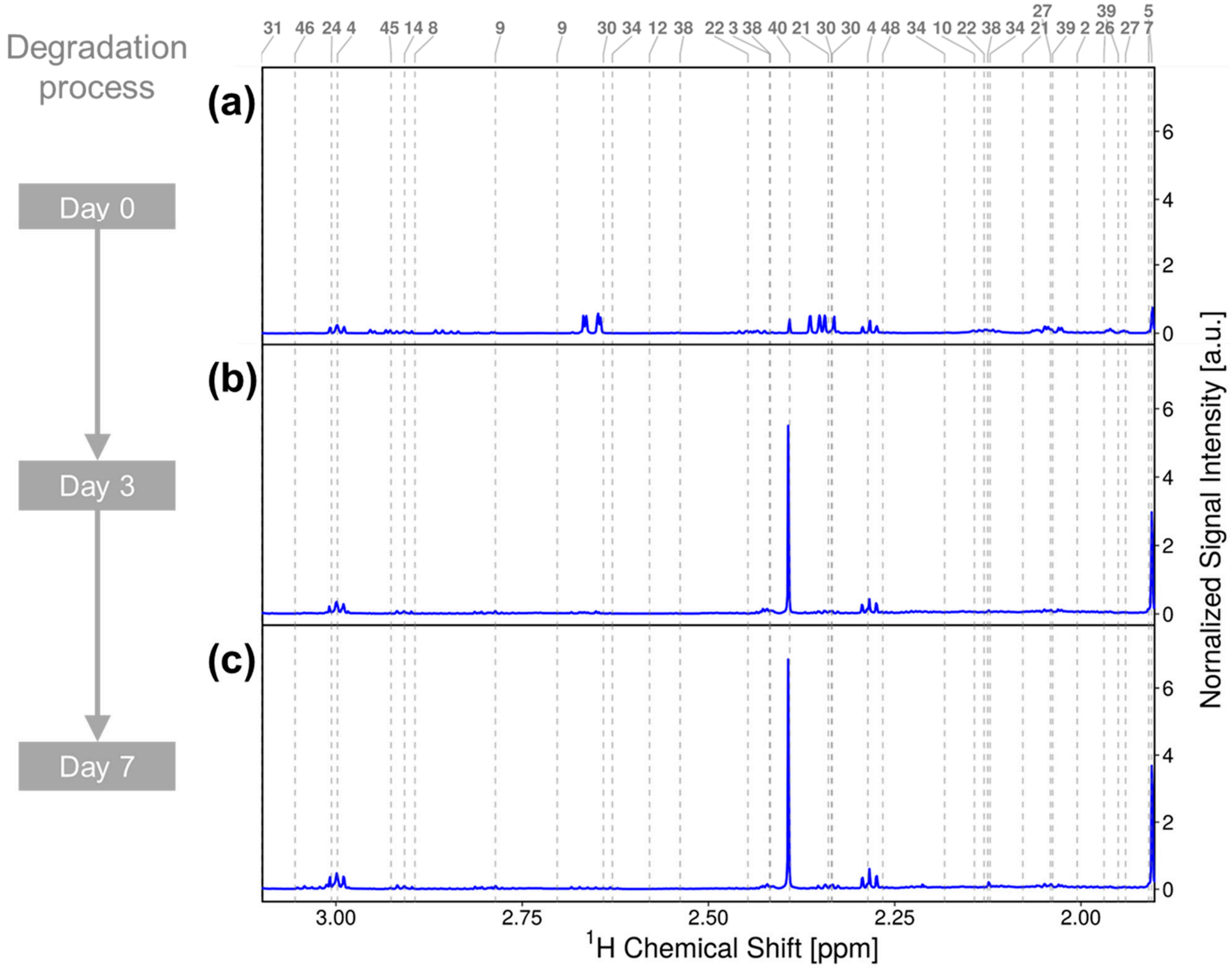

Figure S4. Continued.

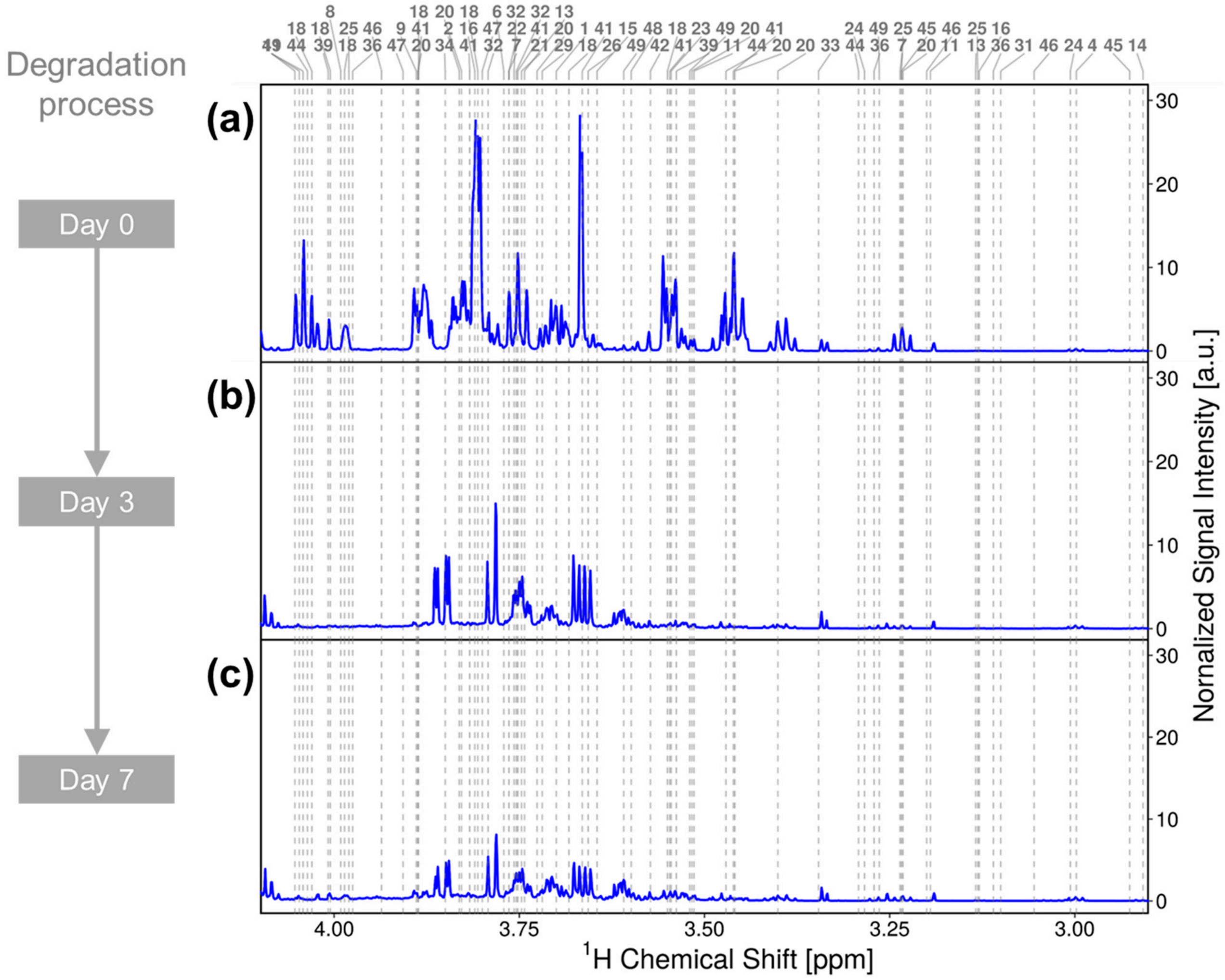

Figure S4. Continued.

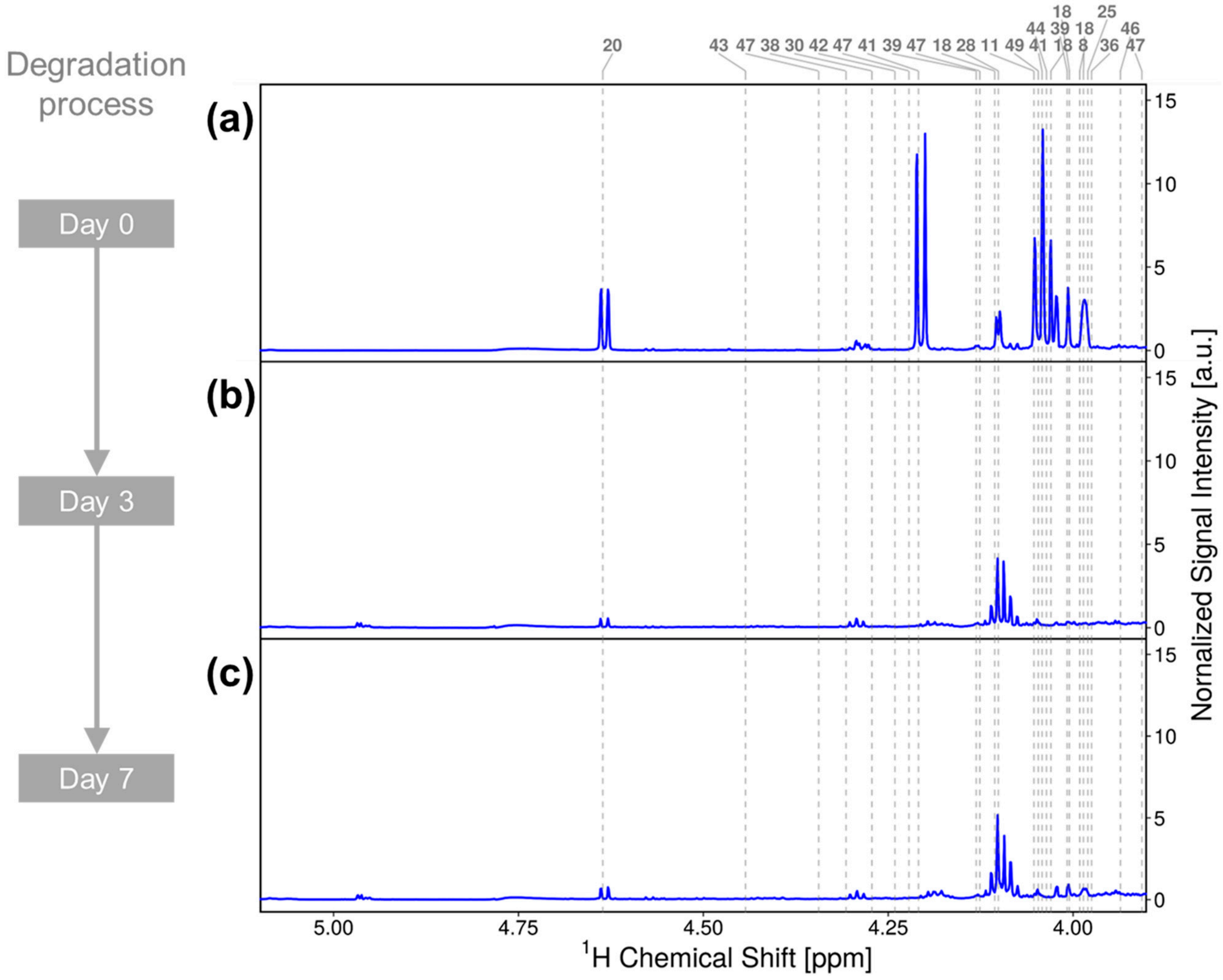

Figure S4. Continued.

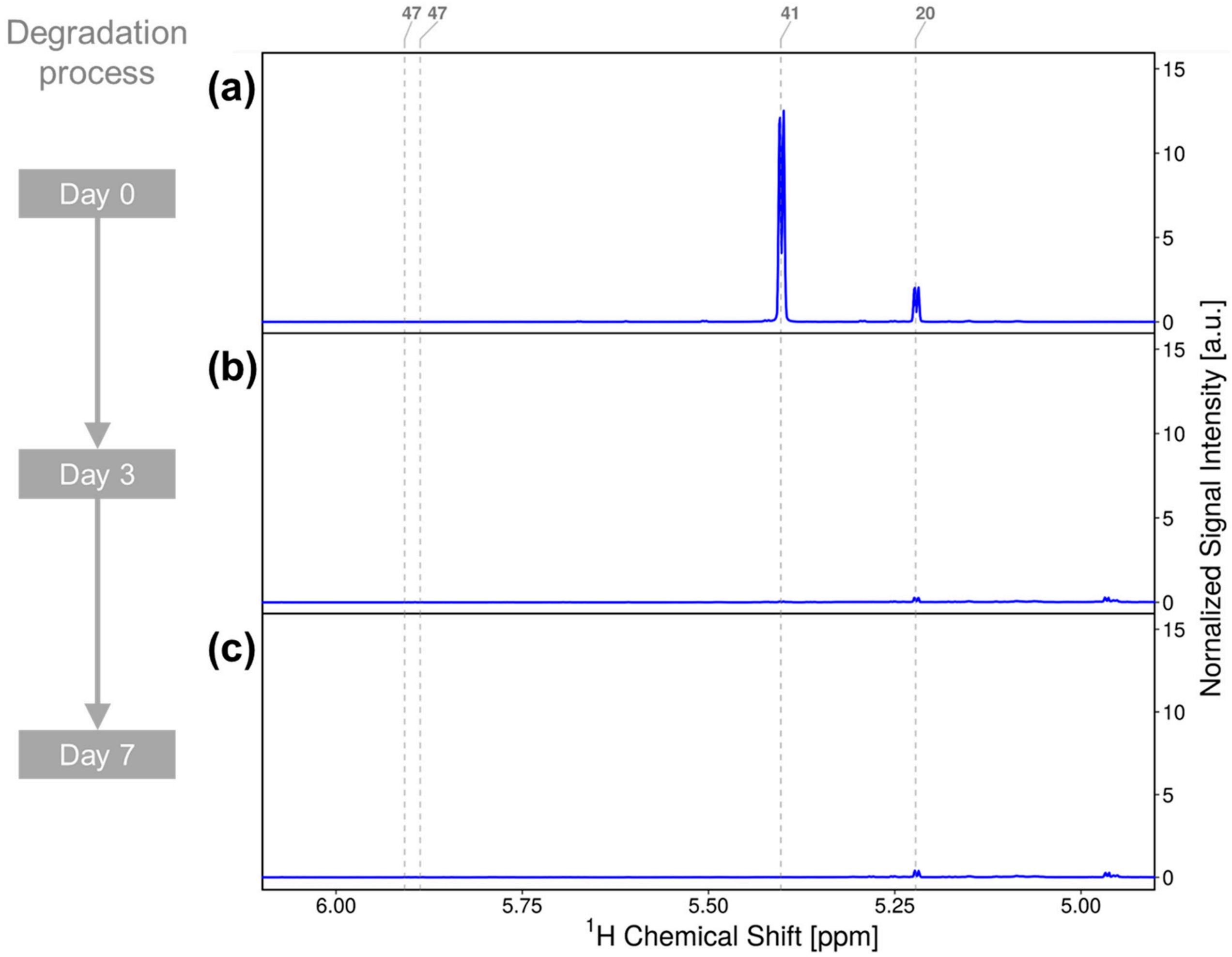

Figure S4. Continued.

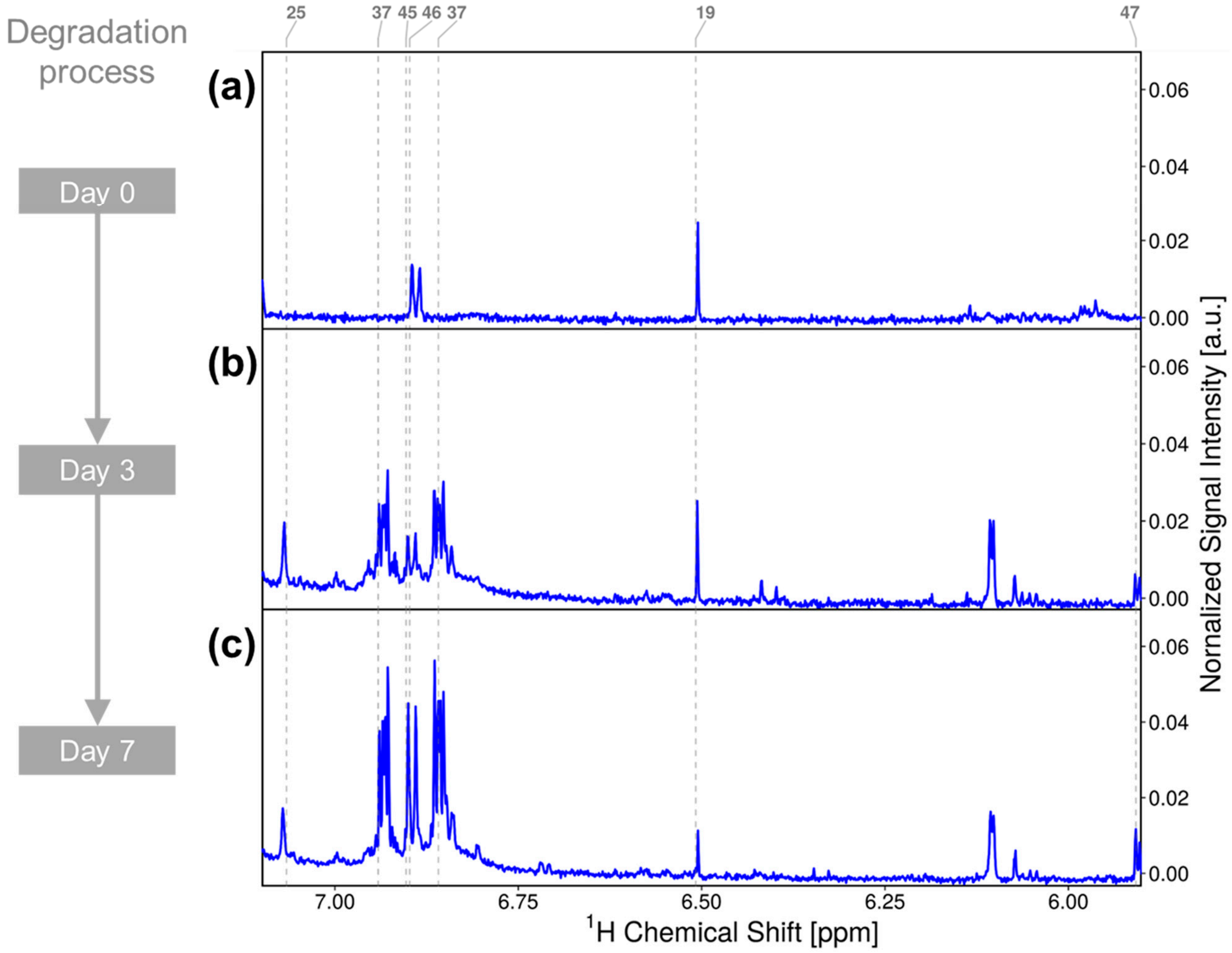

Figure S4. Continued.

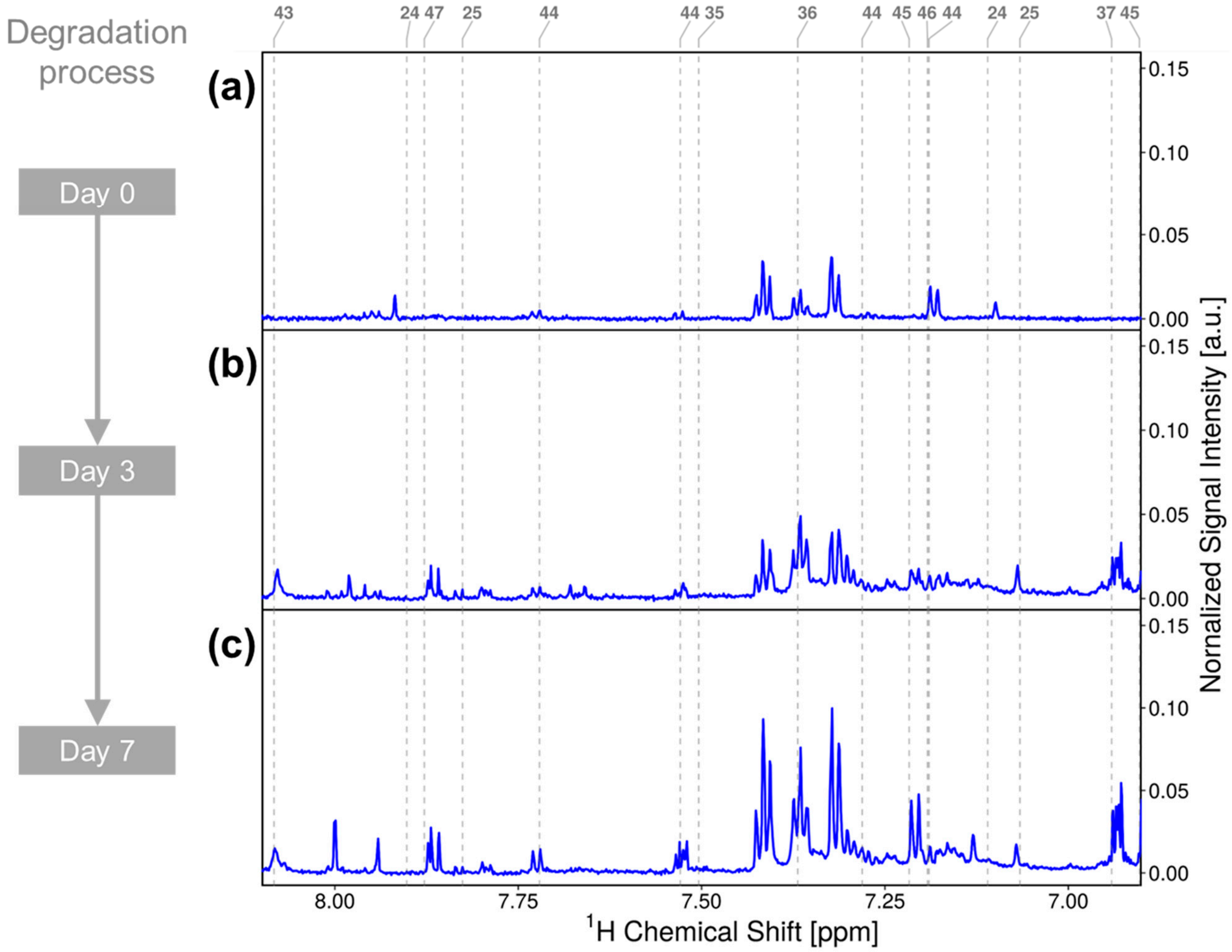

Figure S4. Continued.

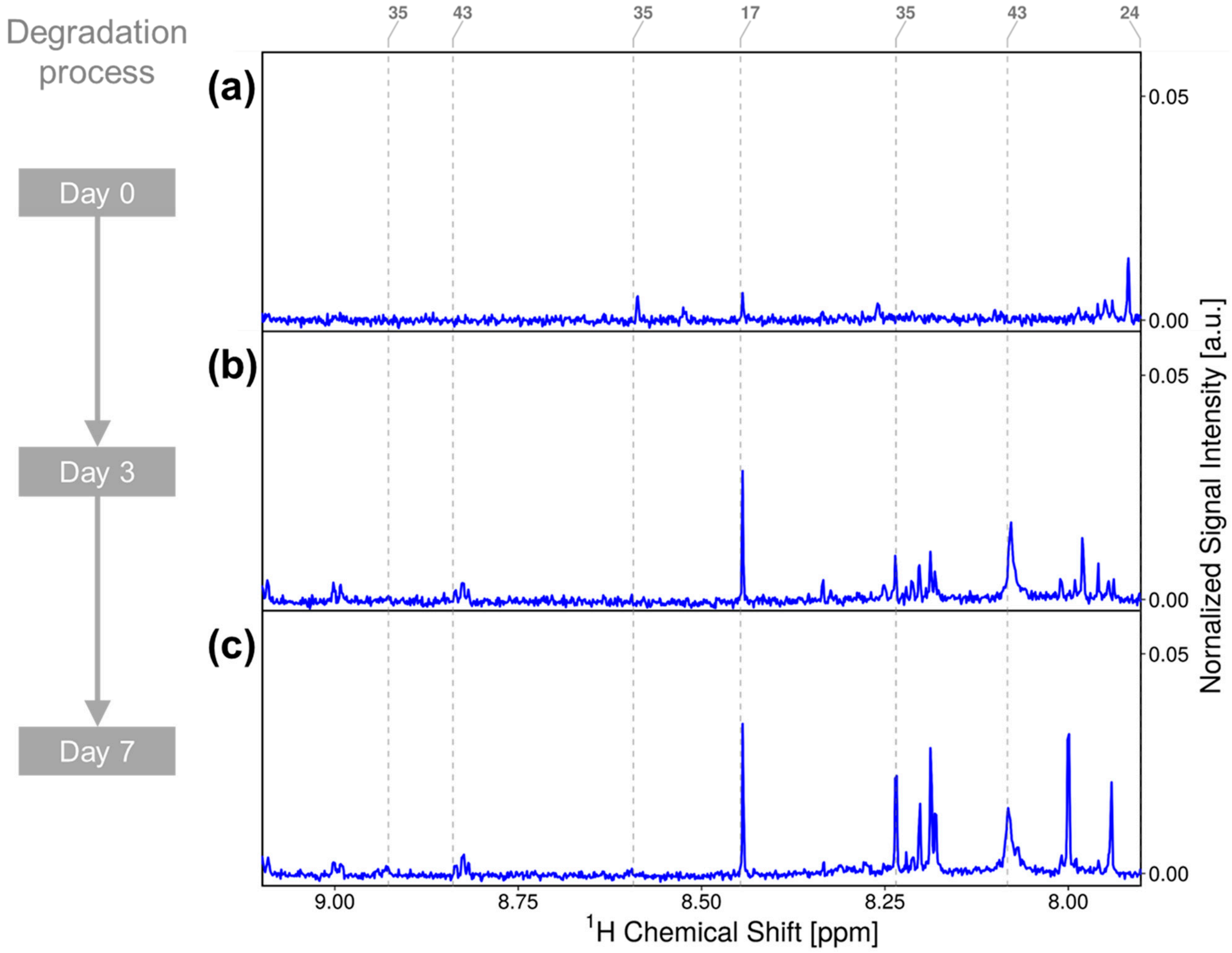

Figure S4. Continued.

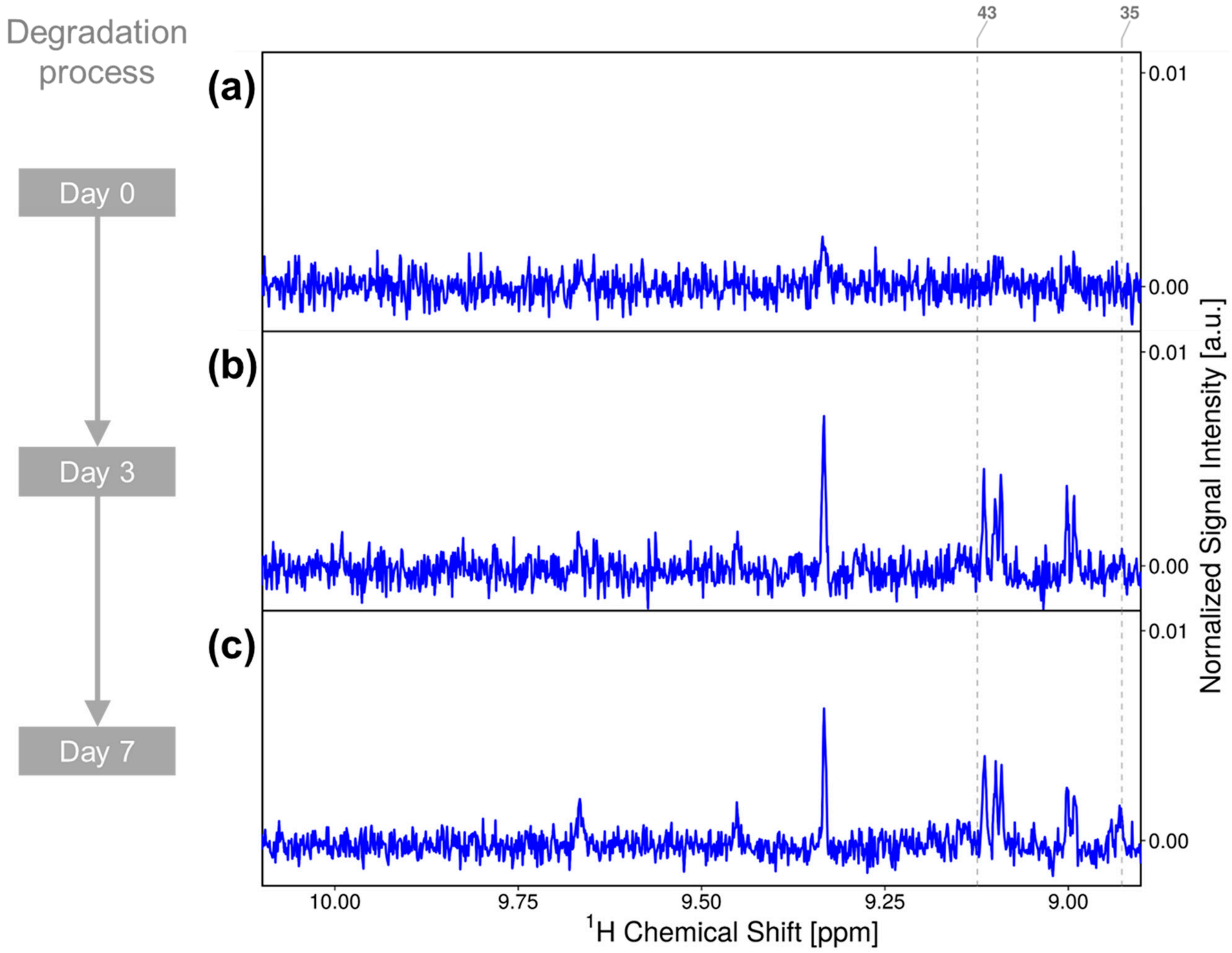

Figure S4. Continued.

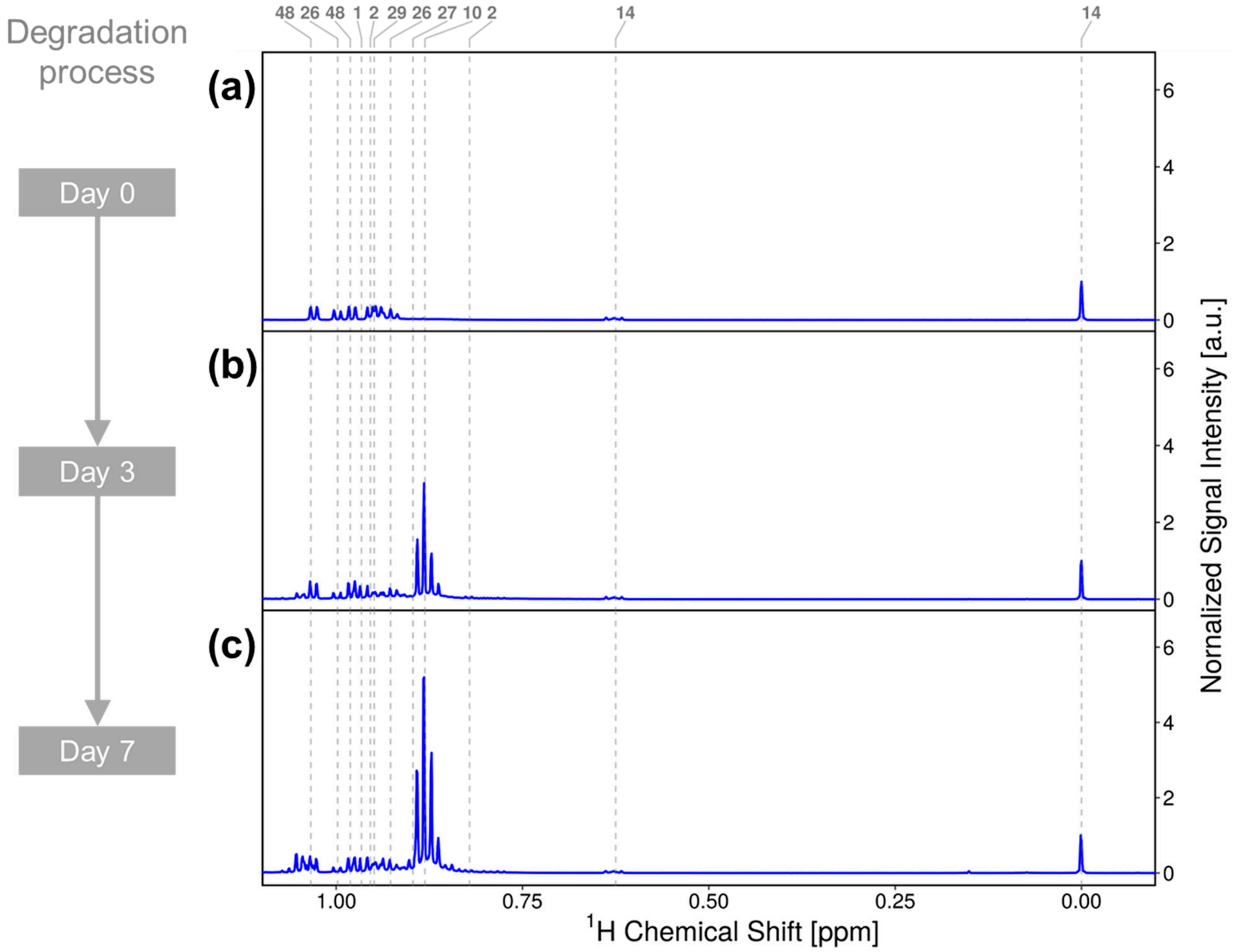

Figure S5. Water presaturated 1D ^{1}H NMR spectra of extracts from (**a**) raw cucumber, Japanese pickled cucumber in rice bran bed on (**b**) day 3 and (**c**) day 7. The values shown at the top of the NMR spectra denote the number of annotated metabolites (1: 2-aminobutyrate; 2: 2-hydroxyisovalerate; 3: 3-hydroxy-3-methylglutarate; 4: 4-aminobutyrate; 5: acetate; 6: alanine; 7: arginine; 8: asparagine; 9: aspartate; 10: butyrate; 11: choline; 12: citrate; 13: citrulline; 14: DSS; 15: ethanol; 16: ethanolamine; 17: formate; 18: fructose; 19: fumarate; 20: glucose; 21: glutamate; 22: glutamine; 23: glycine; 24: histamine; 25: histidine; 26: isoleucine; 27: isovalerate; 28: lactate; 29: leucine; 30: malate; 31: malonate; 32: mannitol; 33: methanol; 34: methionine; 35: nicotinate; 36: phenylalanine; 37: pyrocatechol; 38: pyroglutamate; 39: quinic acid; 40: succinate; 41: sucrose; 42: threonine; 43: trigonelline; 44: tryptophan; 45: tyramine; 46: tyrosine; 47: uridine; 48: valine; and 49: myo-inositol). The dotted lines on the NMR spectra denote chemical shifts of each metabolite registered in BMRB (https://bmrb.io/).

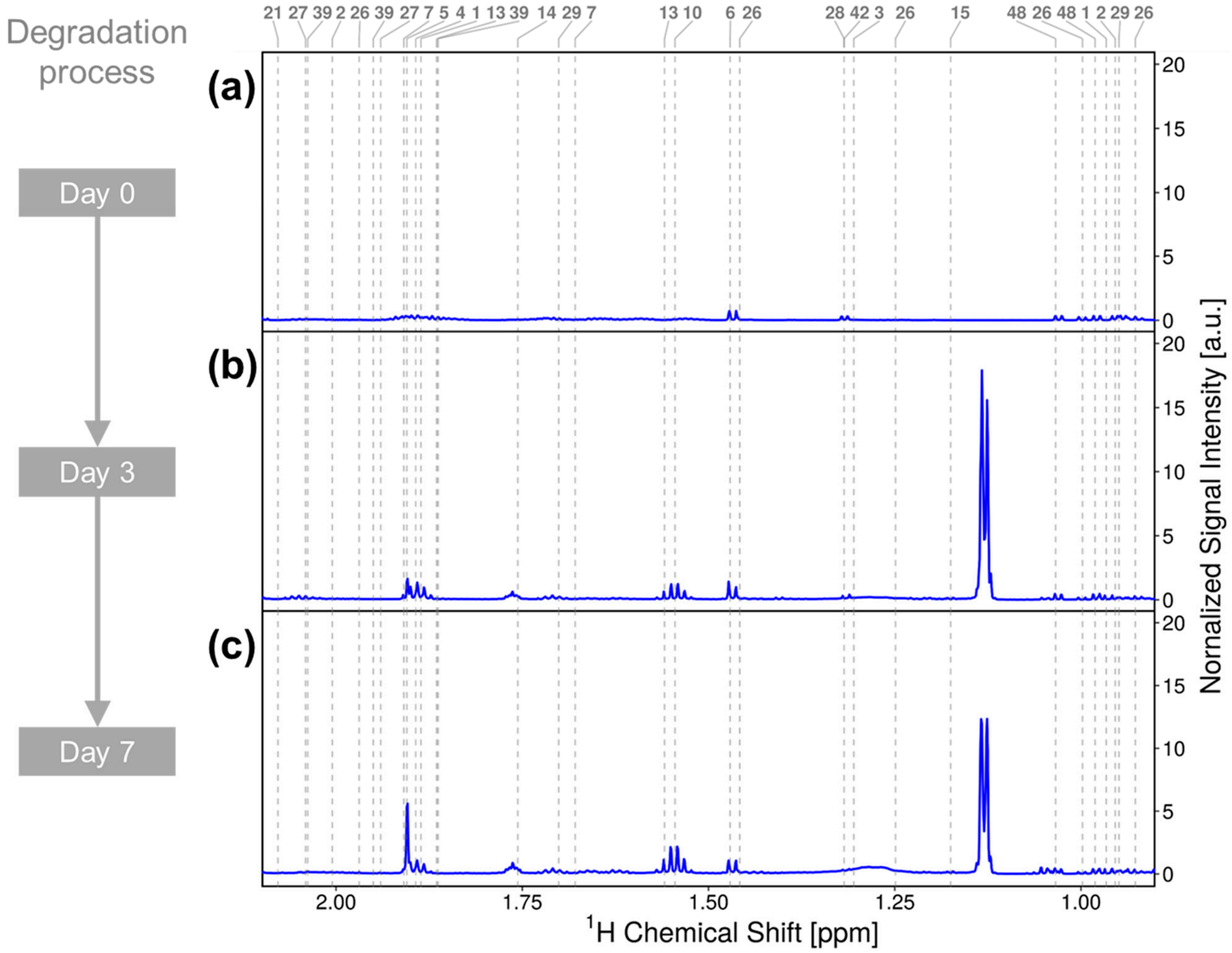

Figure S5. Continued.

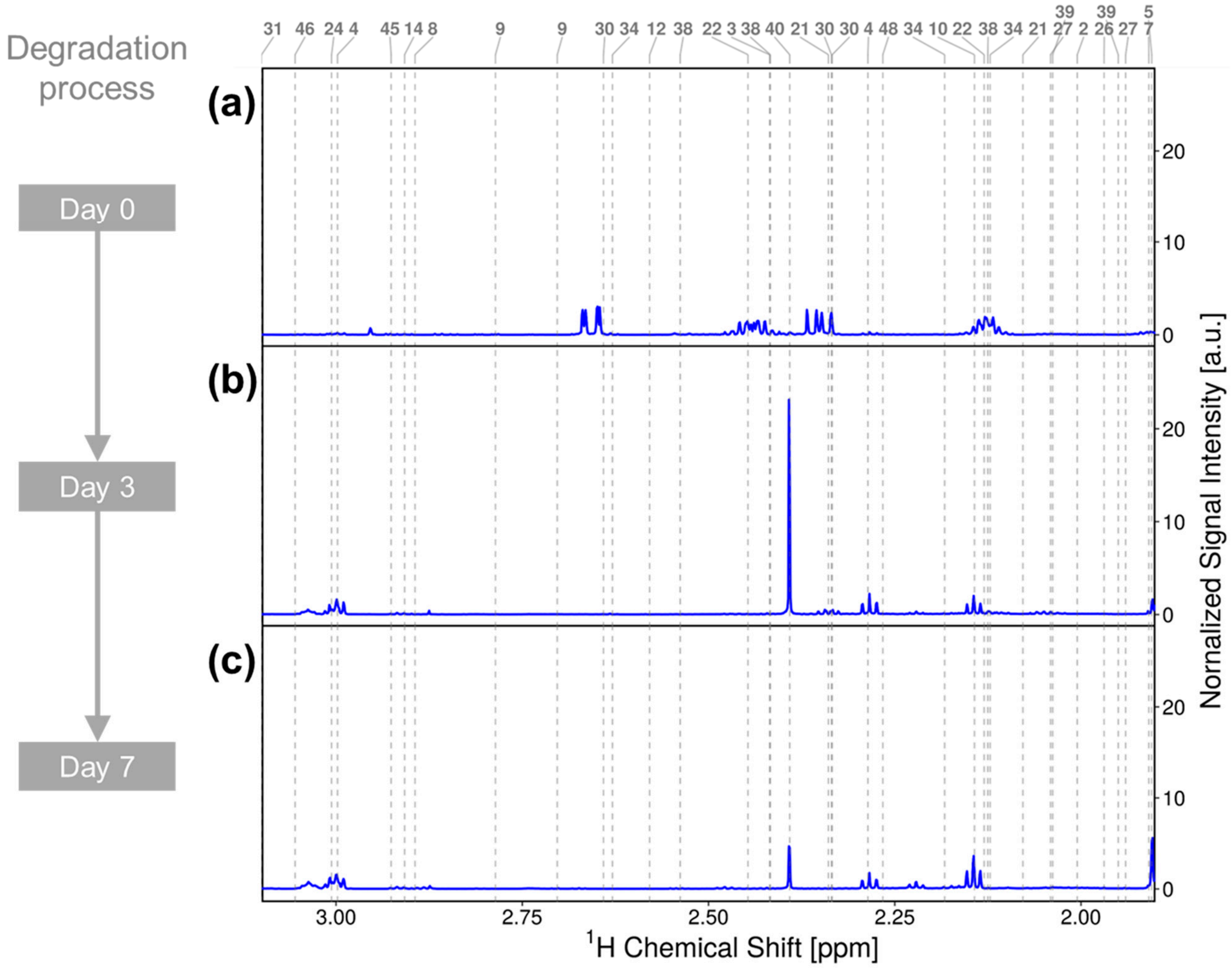

Figure S5. Continued.

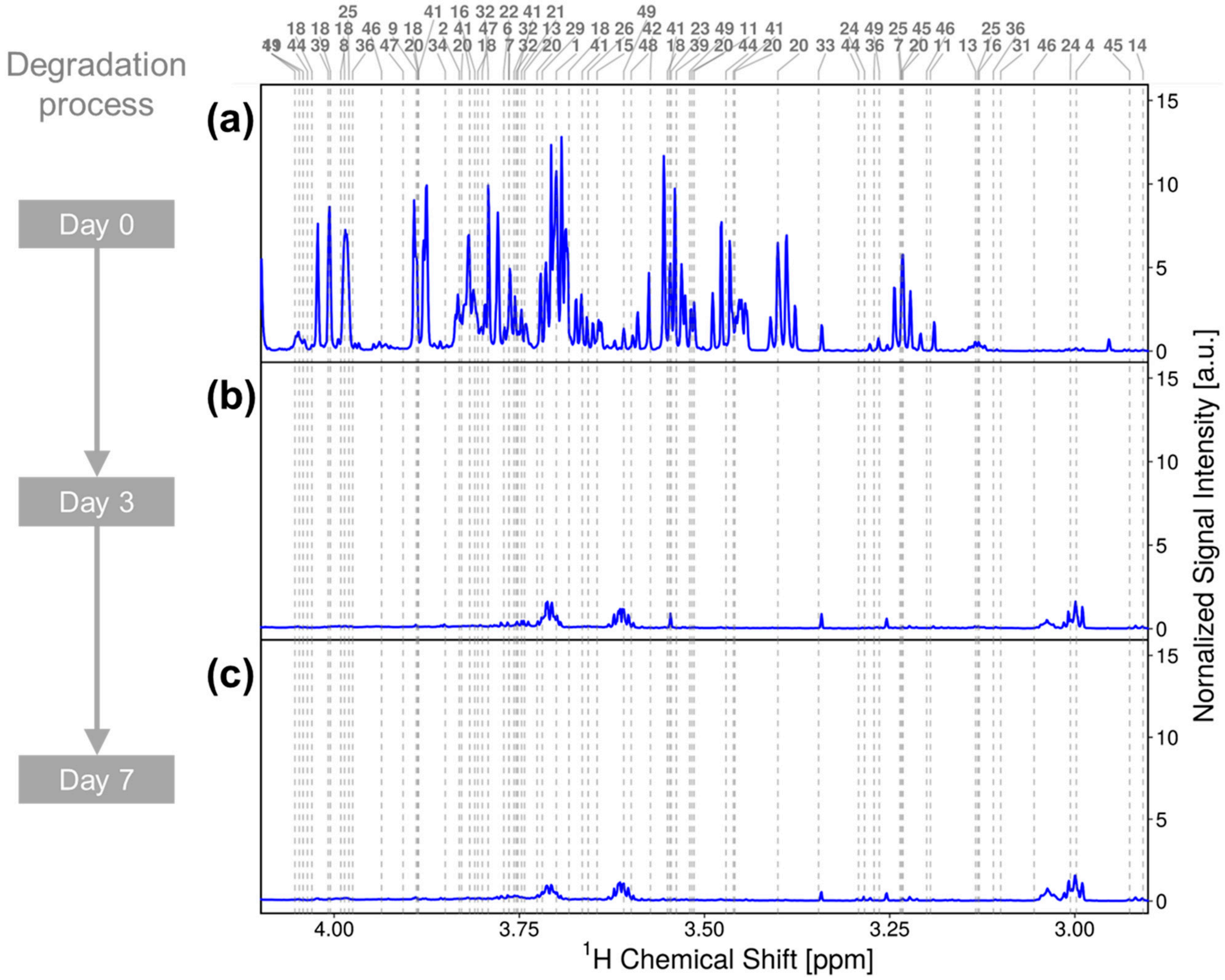

Figure S5. Continued.

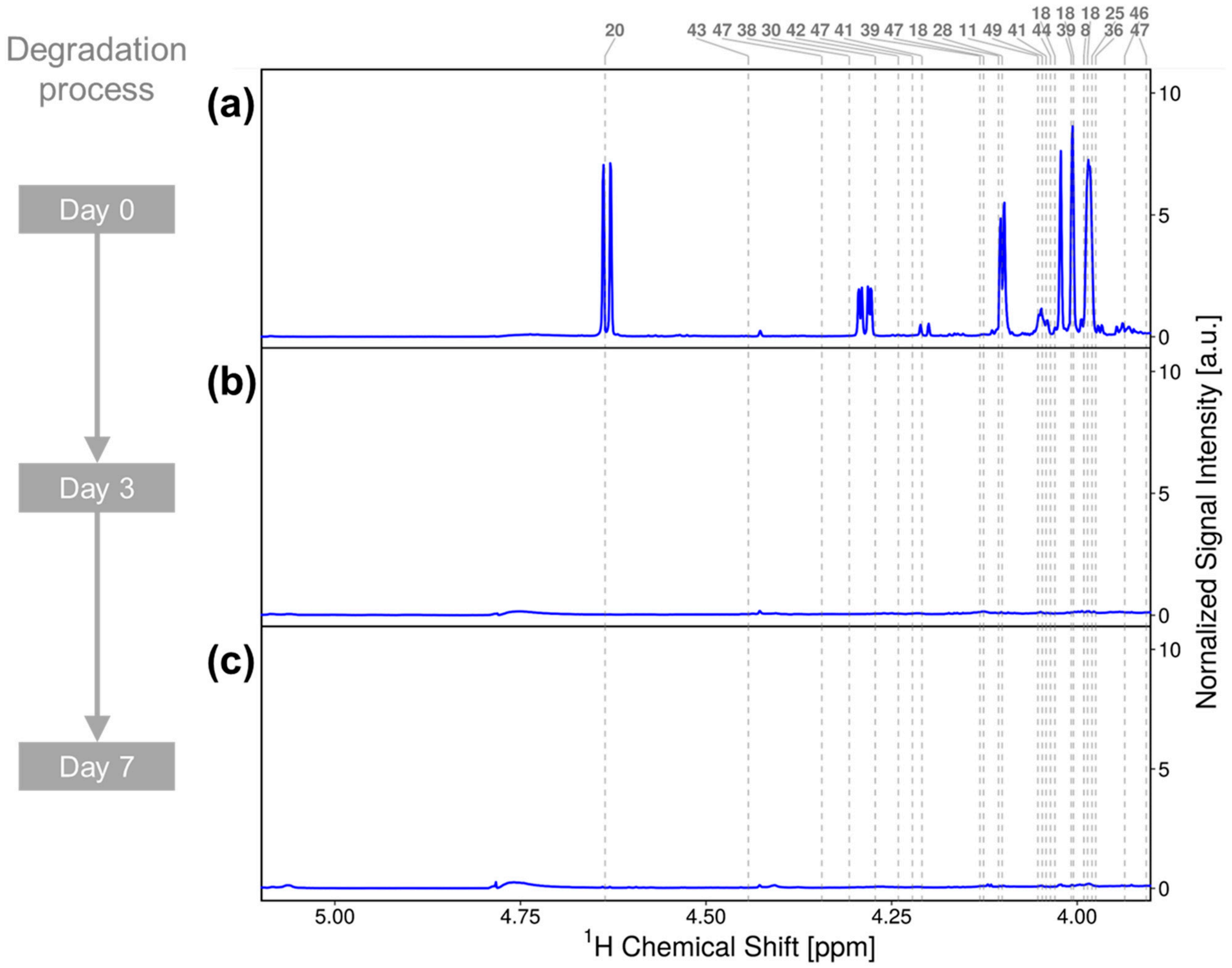

Figure S5. Continued.

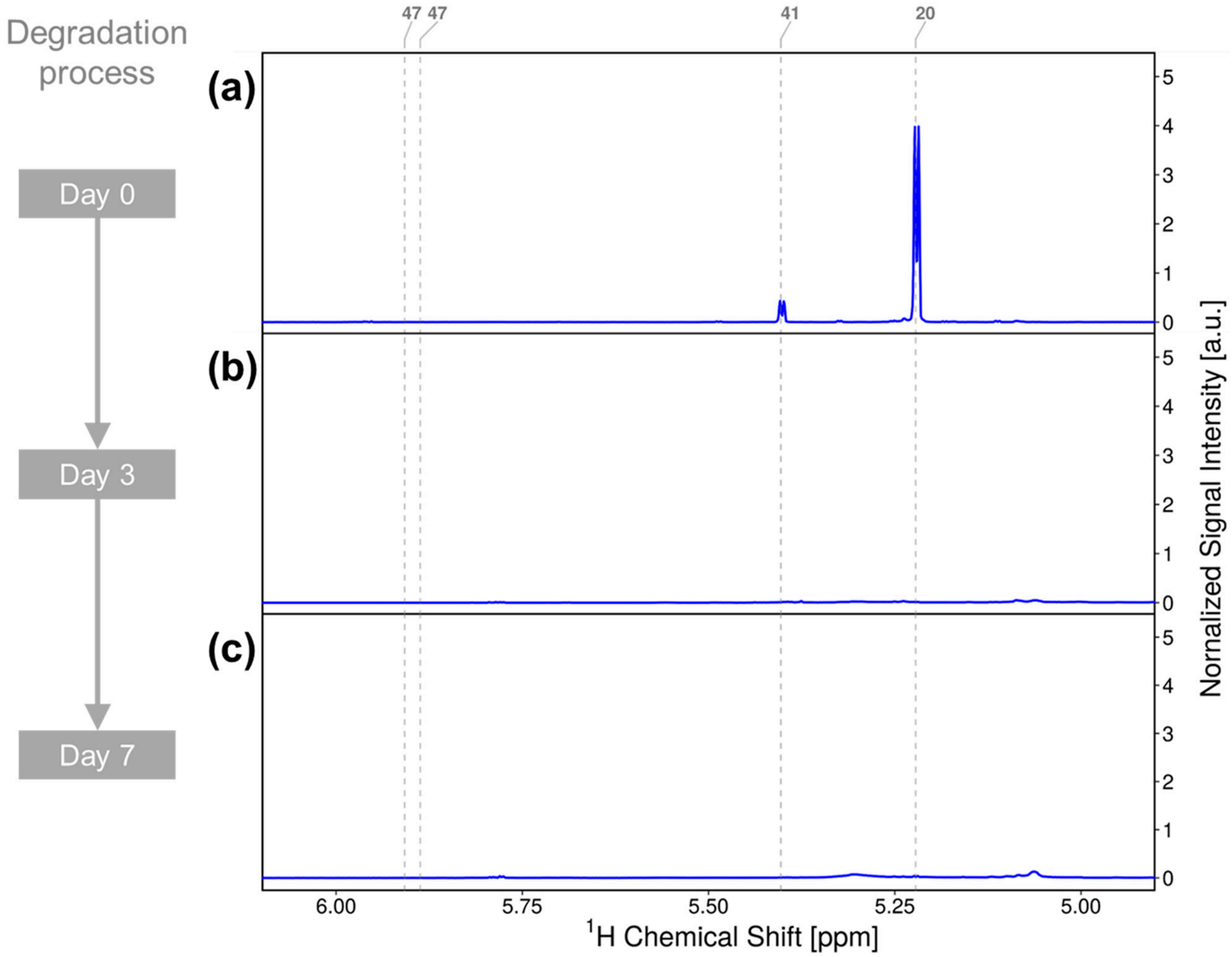

Figure S5. Continued.

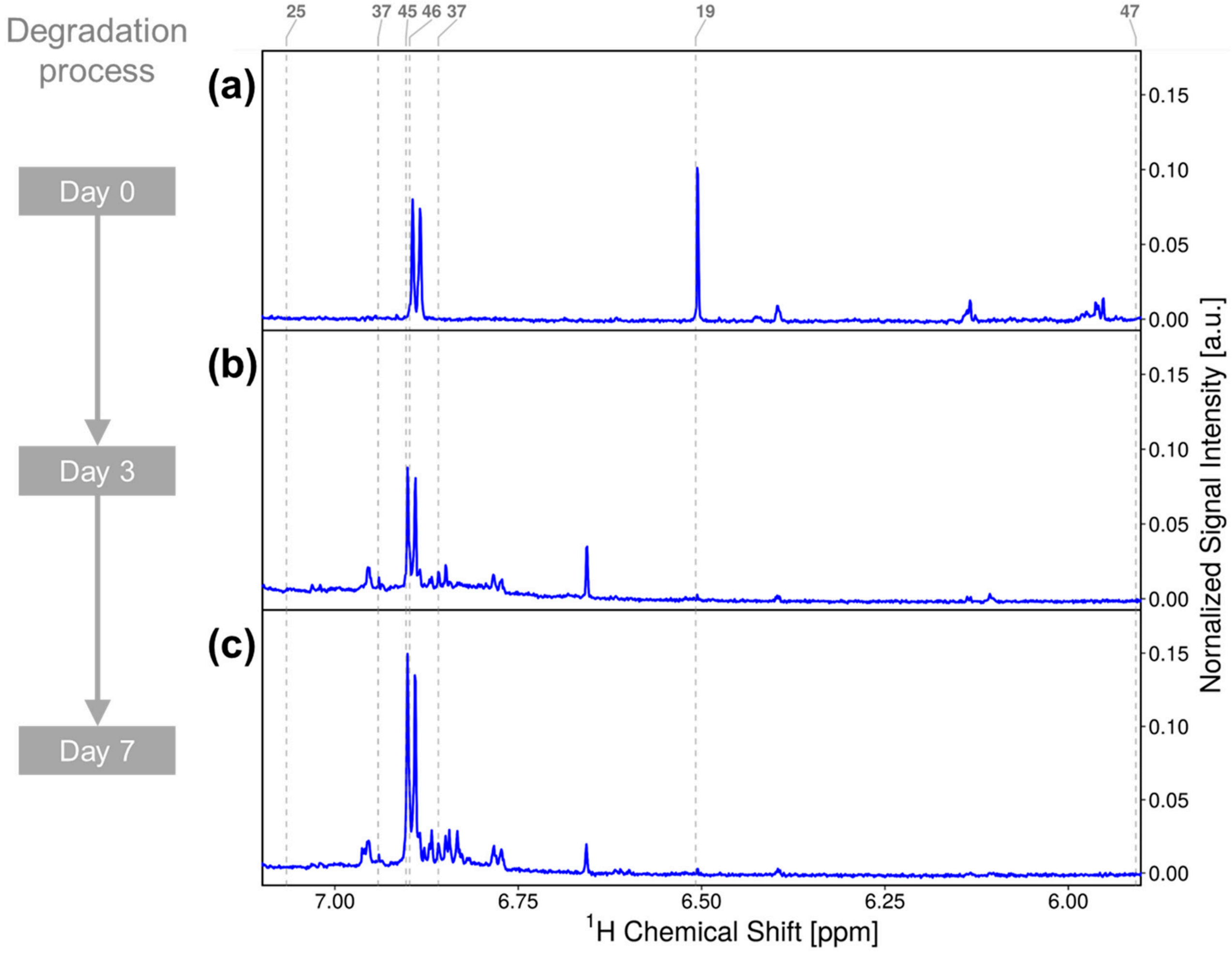

Figure S5. Continued.

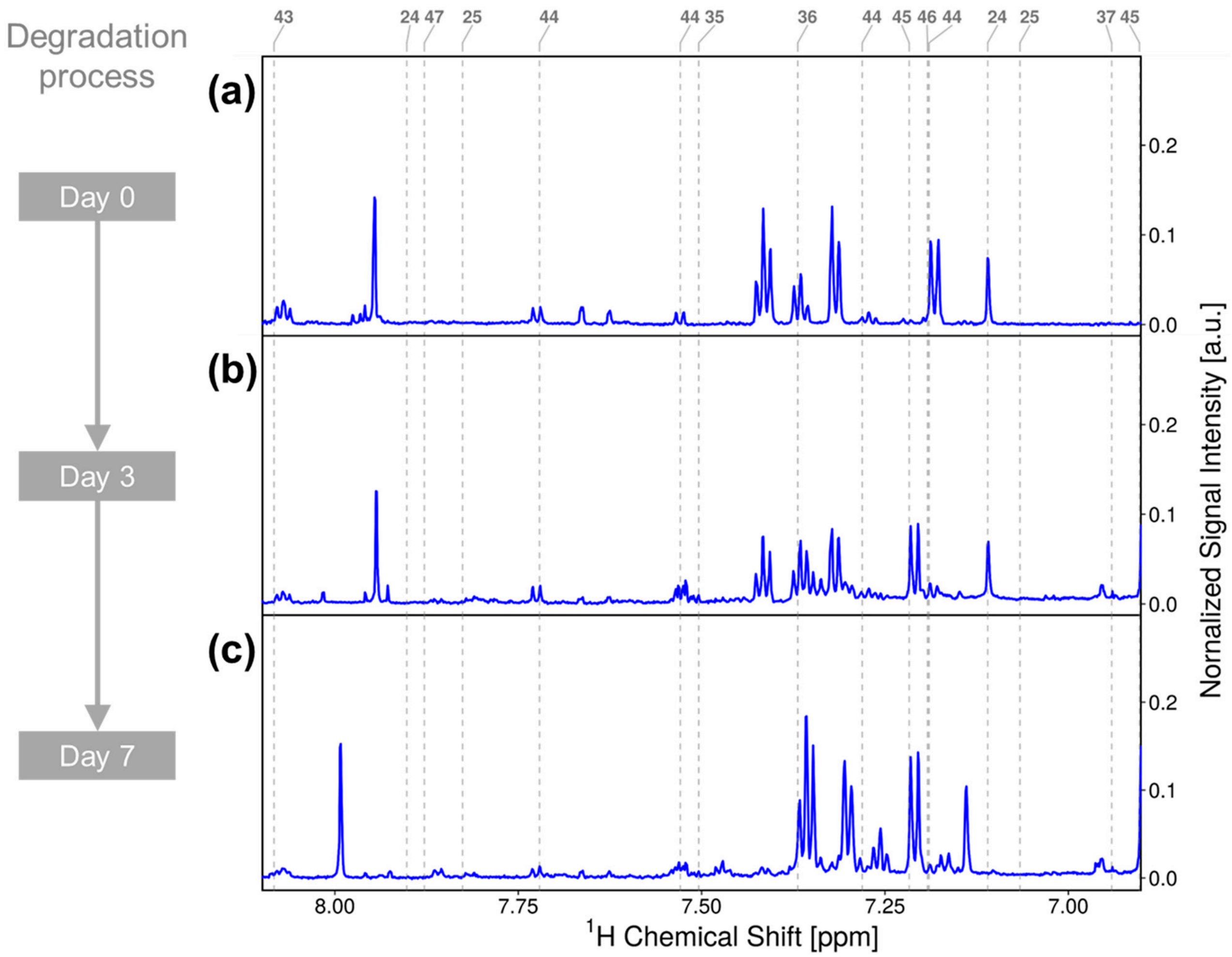

Figure S5. Continued.

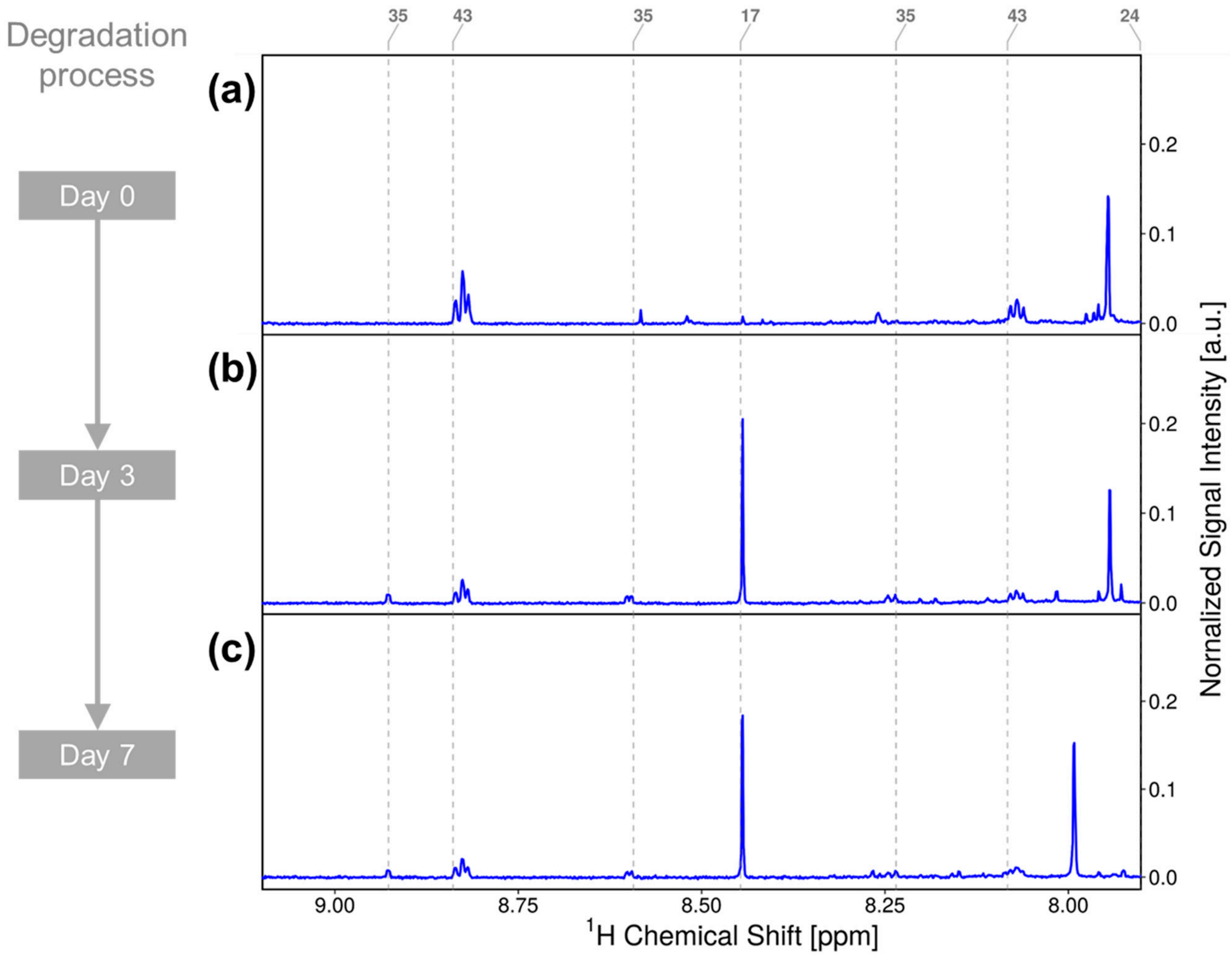

Figure S5. Continued.

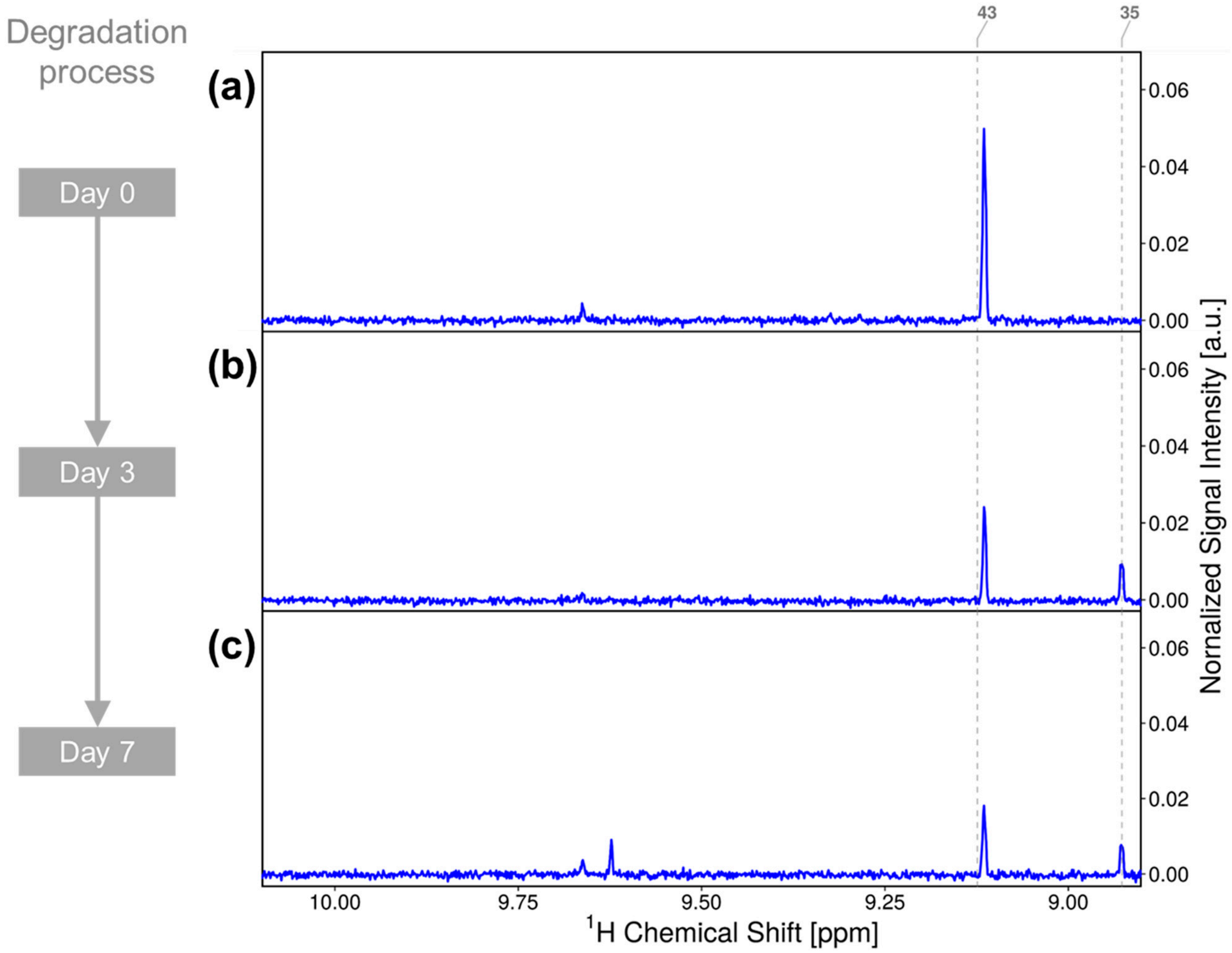

Figure S5. Continued.

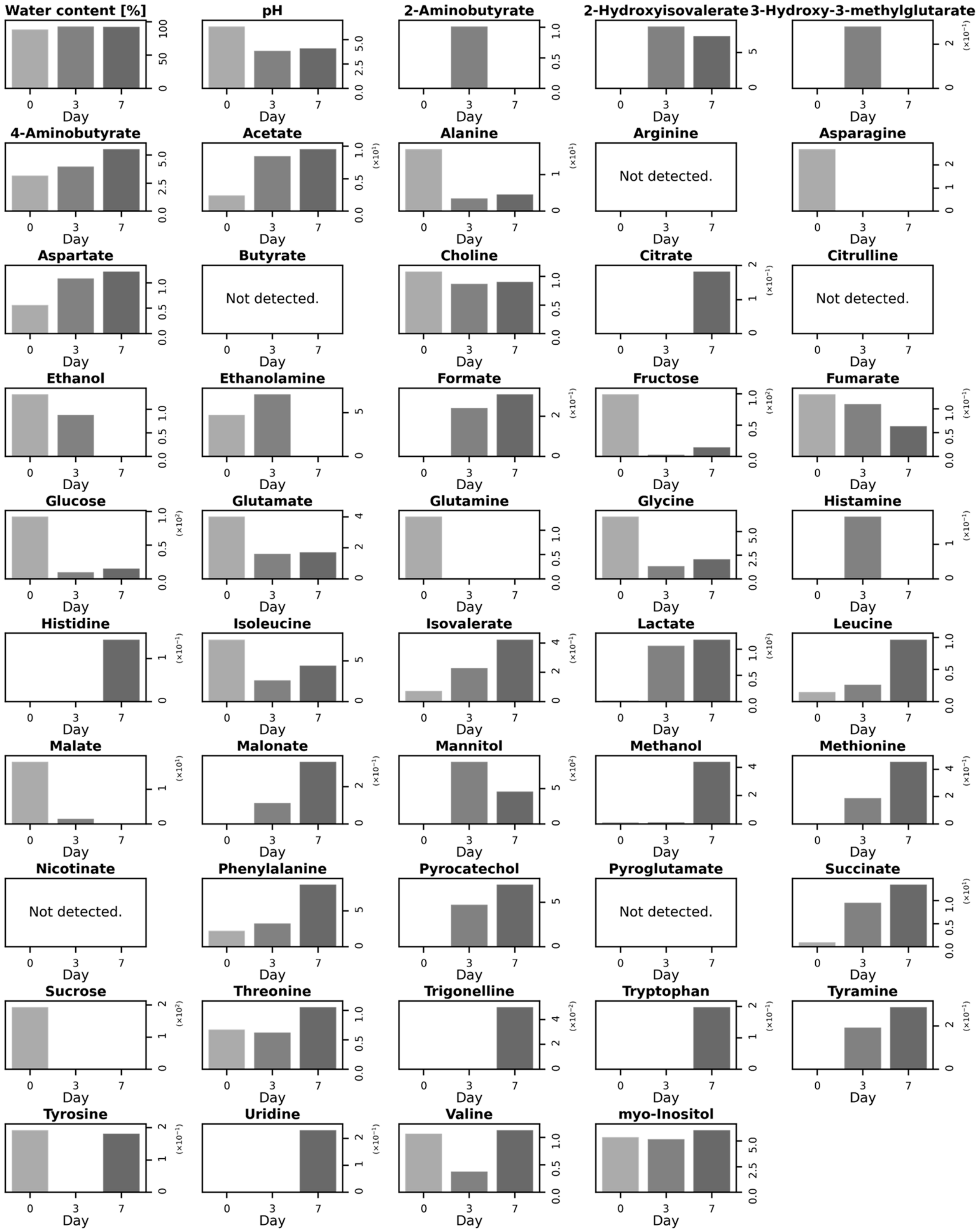

Figure S6. The transition of water content and pH in rice bran bed, and the amount of each metabolite in carrot on each day of degradation. The amount of each metabolite was estimated with the Chenomx NMR Suite software using water presaturated 1D ^{1}H NMR spectra shown in Figure S4, and expressed as a relative ratio in comparison to DSS.

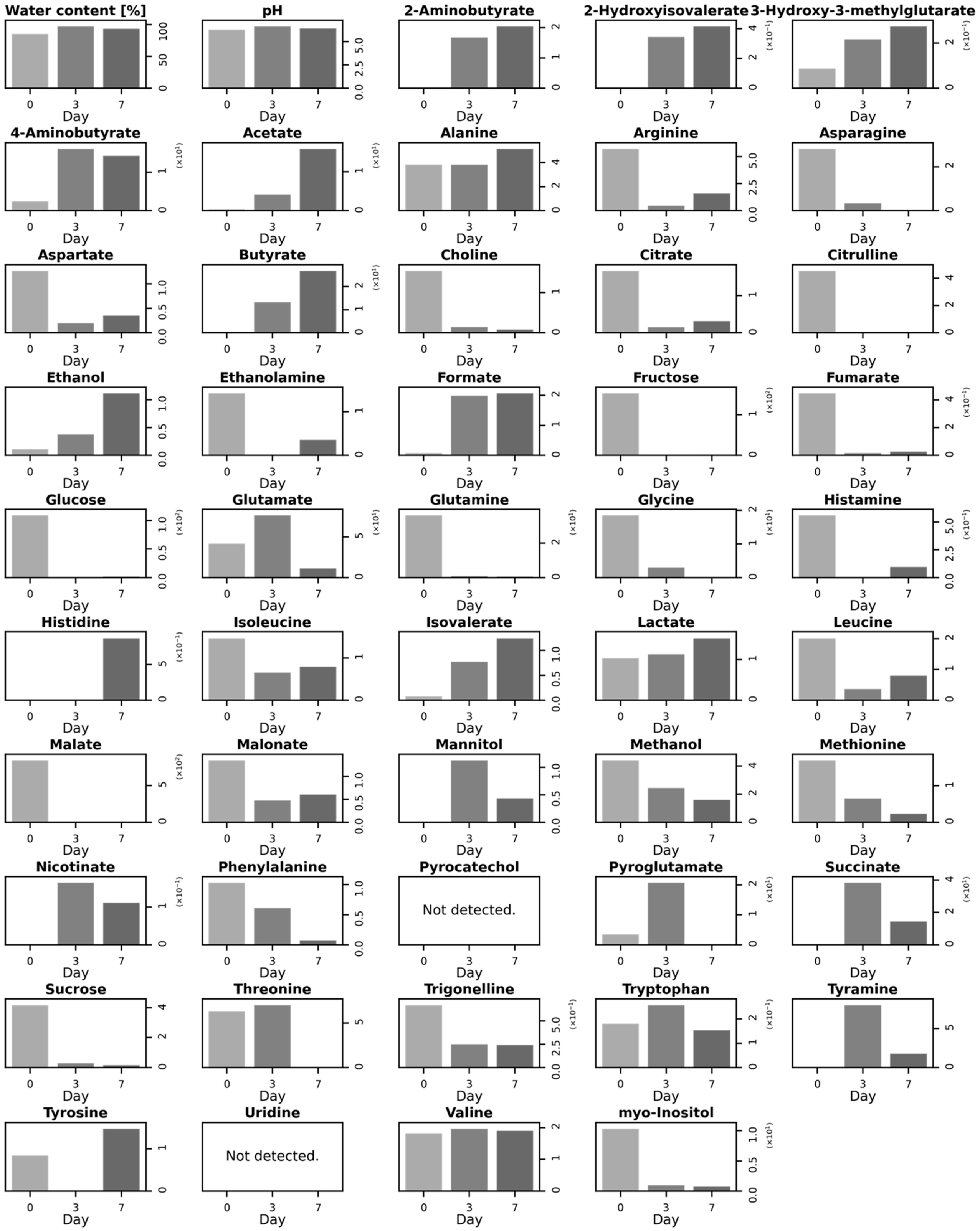

Figure S7. The transition of water content and pH in rice bran bed, and the amount of each metabolite in cucumber on each day of degradation. The amount of each metabolite was estimated with the Chenomx NMR Suite software using water presaturated 1D ^{1}H NMR spectra shown in Figure S5, and expressed as a relative ratio in comparison to DSS.

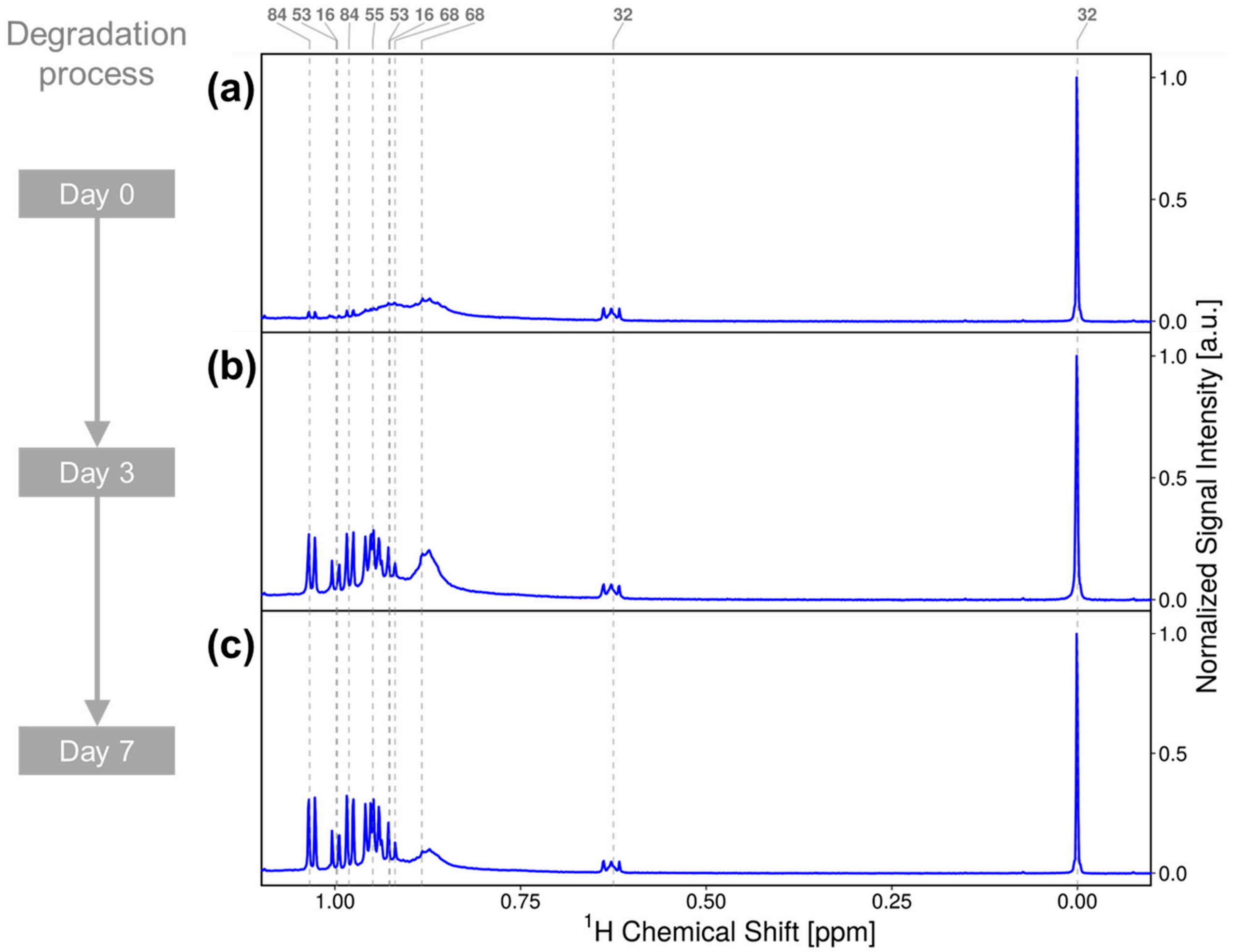

Figure S8. Water presaturated 1D ^{1}H NMR spectra of rice bran extracts on (**a**) day 0, (**b**) day 3, and (**c**) day 7 in the control experiment. The values at the top of the NMR spectra denote number of annotated metabolites (1: 1,3-dihydroxyacetone; 2: 1,3-dimethylurate; 3: 1,7-dimethylxanthine; 4: 2'-deoxyuridine; 5: 2-aminoadipate; 6: 2-phenylpropionate; 7: 2-phosphoglycerate; 8: 3-hydroxybutyrate; 9: 3-hydroxyisovalerate; 10: 3-hydroxykynurenine; 11: 4-aminobutyrate; 12: acetaminophen; 13: acetate; 14: adenosine; 15: alanine; 16: alloisoleucine; 17: anserine; 18: arabinose; 19: arginine; 20: asparagine; 21: aspartate; 22: betaine; 23: carnosine; 24: cellobiose; 25: choline; 26: citraconate; 27: citrate; 28: creatine; 29: creatine phosphate; 30: cystine; 31: cytidine; 32: DSS; 33: ethanol; 34: ethanolamine; 35: ethylene glycol; 36: formate; 37: fructose; 38: fucose; 39: galactarate; 40: galactitol; 41: galactose; 42: gluconate; 43: glucose; 44: glucuronate; 45: glutamine; 46: glycerate; 47: glycerol; 48: glycine; 49: glycolate; 50: histamine; 51: homoserine; 52: imidazole; 53: isoleucine; 54: lactate; 55: leucine; 56: lysine; 57: malonate; 58: maltose; 59: methanol; 60: methionine; 61: *N*-acetylaspartate; 62: *N*-acetylserotonin; 63: *N*-acetyltyrosine; 64: *N*-phenylacetylglycine; 65: *O*-acetylcholine; 66: *O*-phosphocholine; 67: ornithine; 68: pantothenate; 69: phenylalanine; 70: pimelate; 71: proline; 72: propylene glycol; 73: riboflavin; 74: sarcosine; 75: succinate; 76: sucrose; 77: threonine; 78: thymol; 79: trehalose; 80: trigonelline; 81: trimethylamine; 82: tyrosine; 83: uridine; 84: valine; 85: vanillate; 86: xanthine; 87: xylose; 88: myo-inositol; 89: *sn*-glycero-3-phosphocholine; and 90: π-methylhistidine). The dotted lines on the NMR spectra denote the chemical shifts of each metabolite registered in BMRB (https://bmrb.io/).

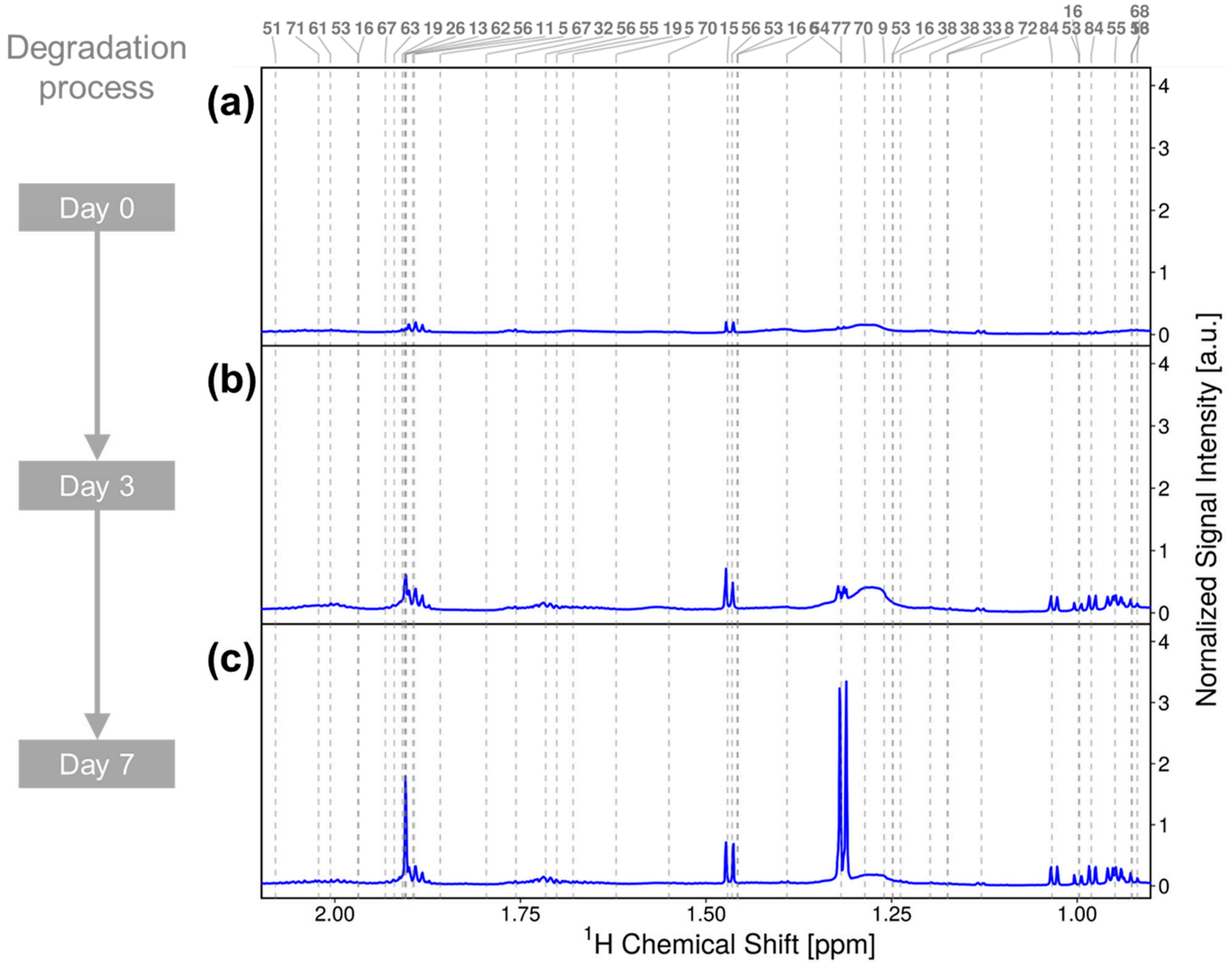

Figure S8. Continued.

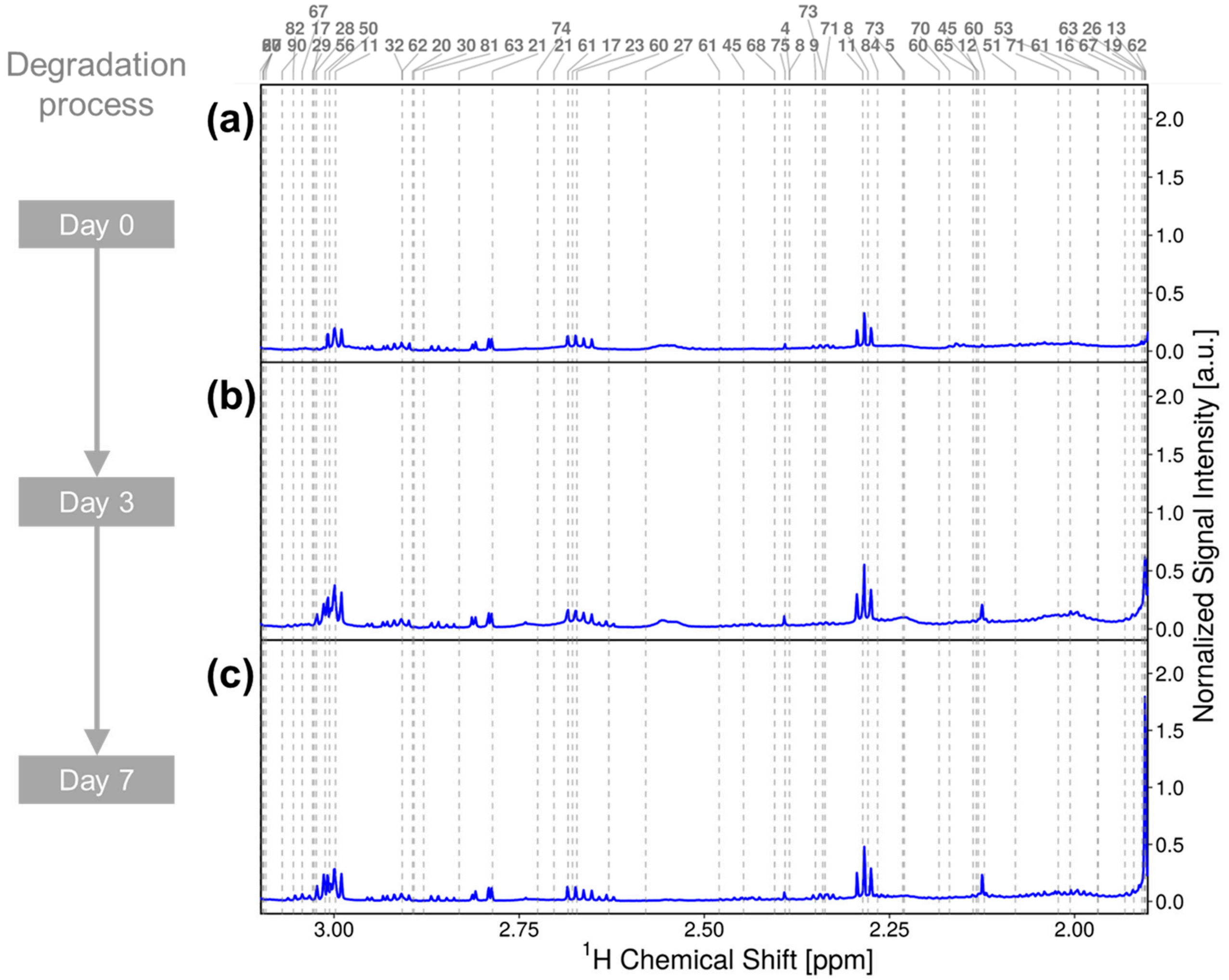

Figure S8. Continued.

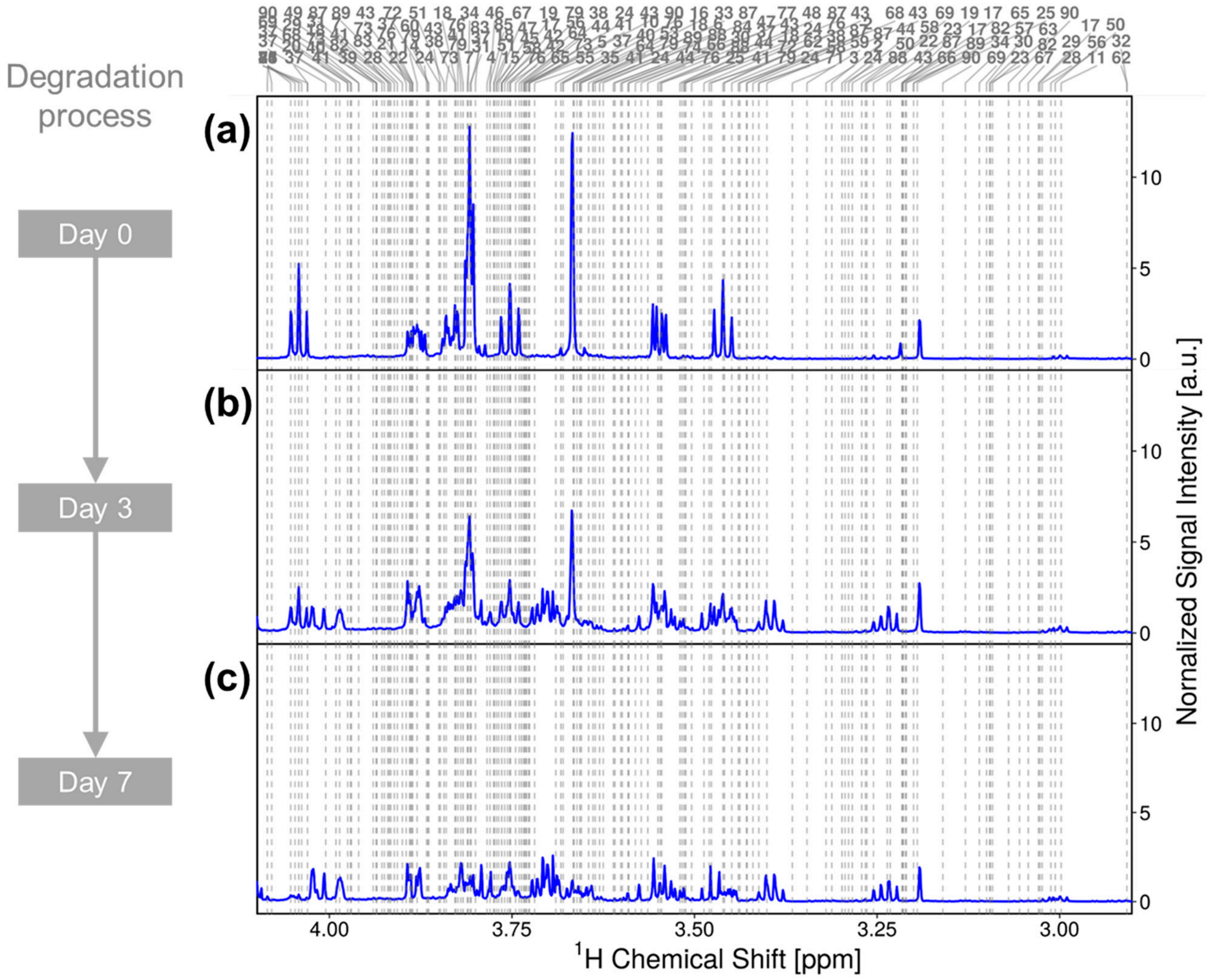

Figure S8. Continued.

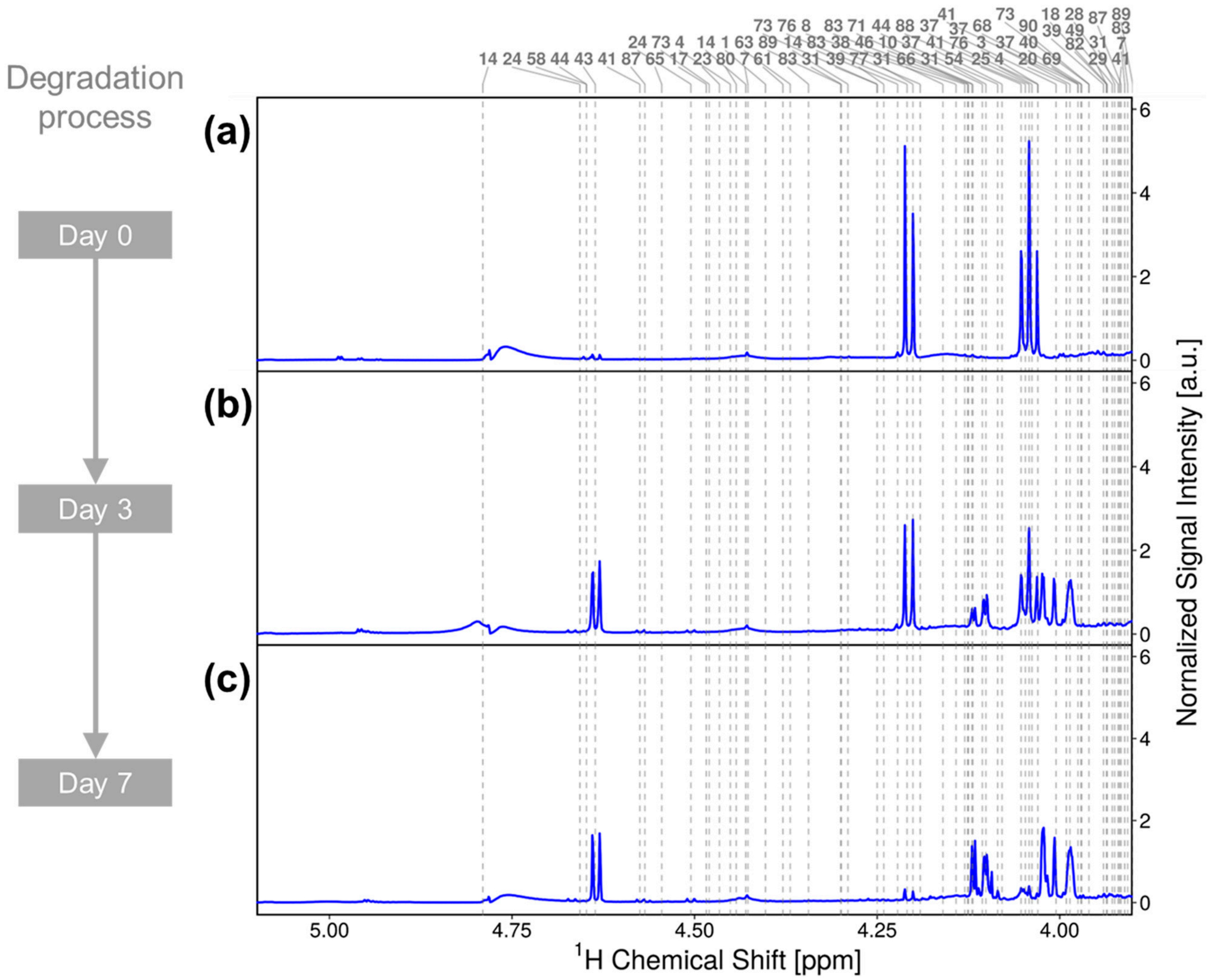

Figure S8. Continued.

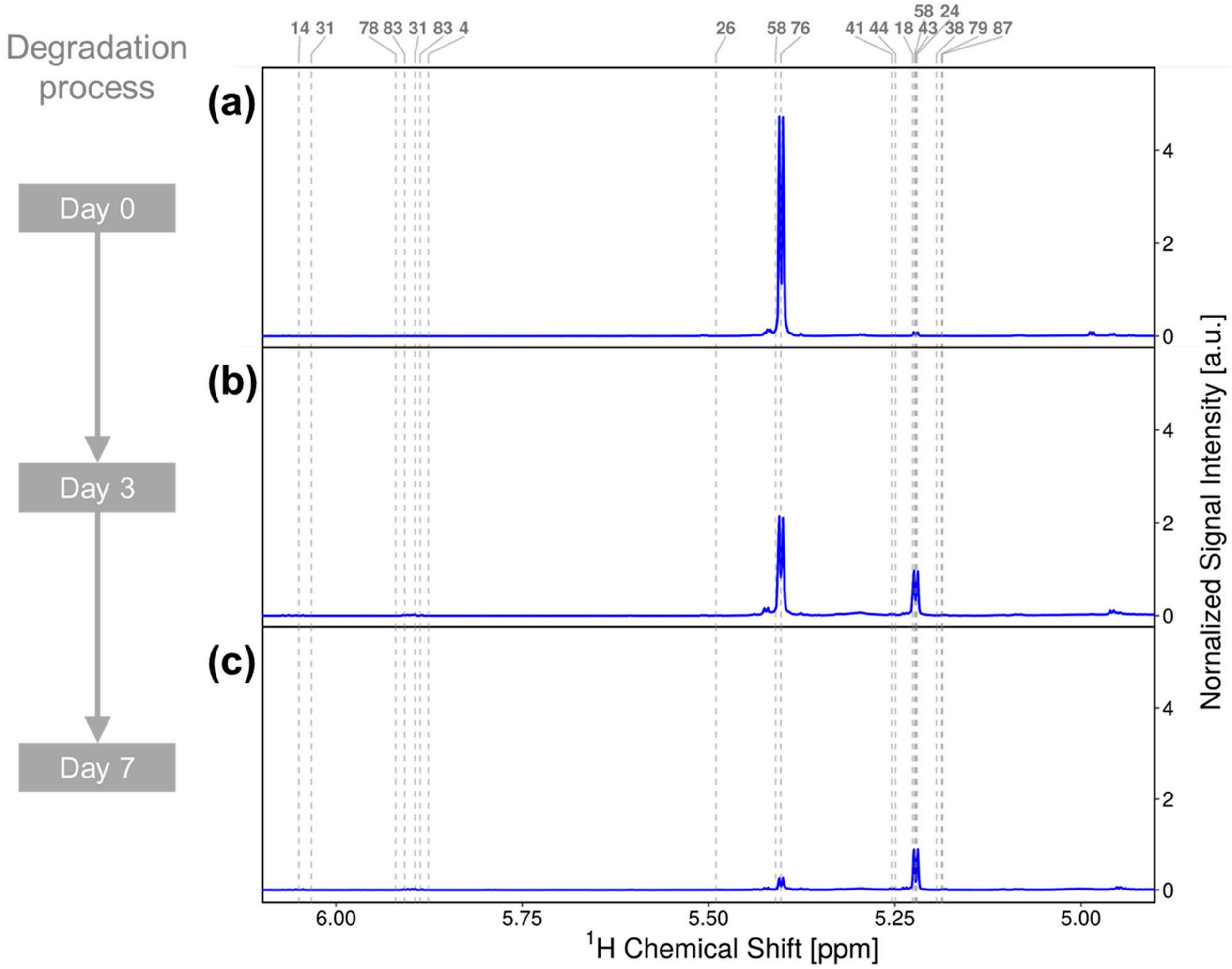

Figure S8. Continued.

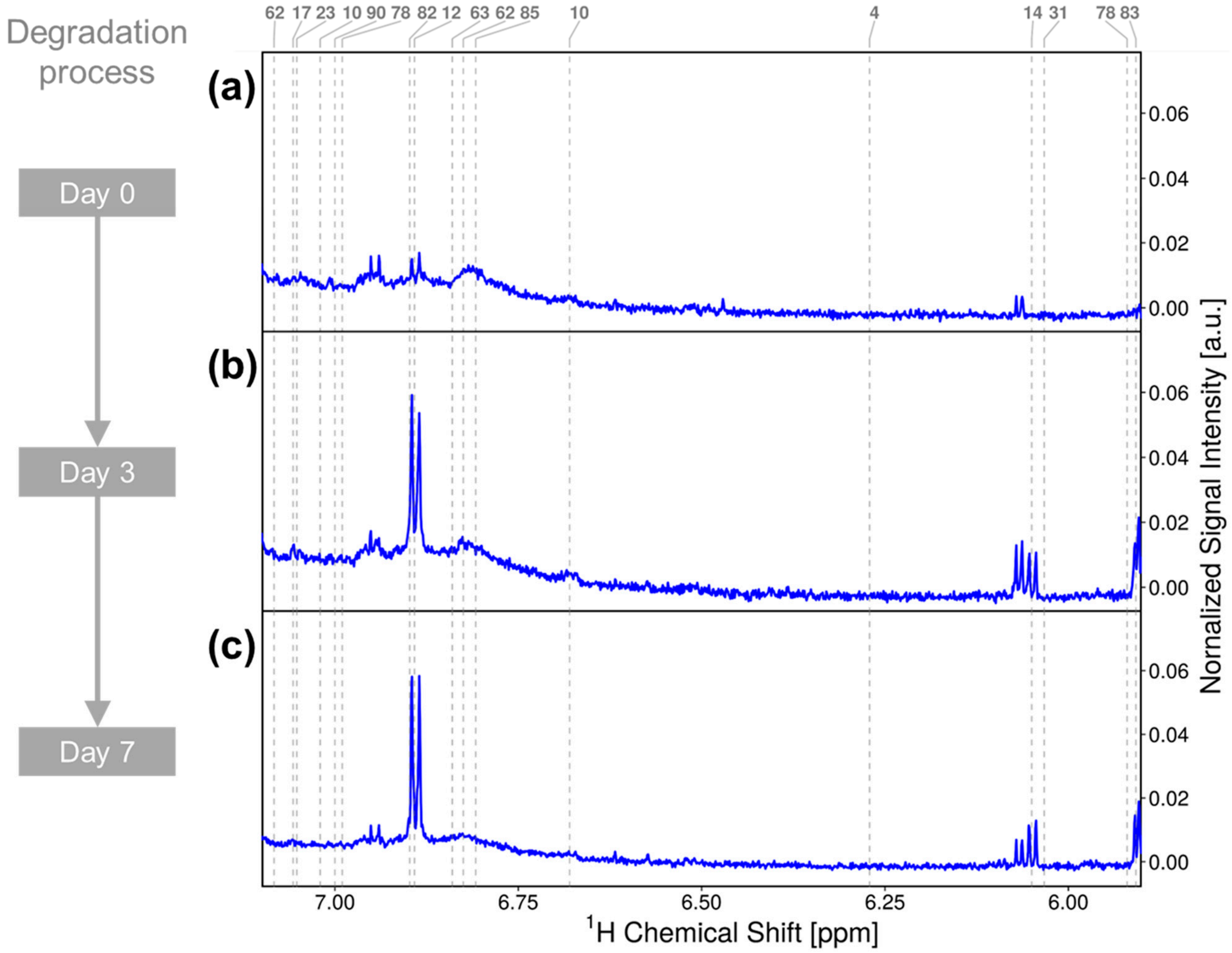

Figure S8. Continued.

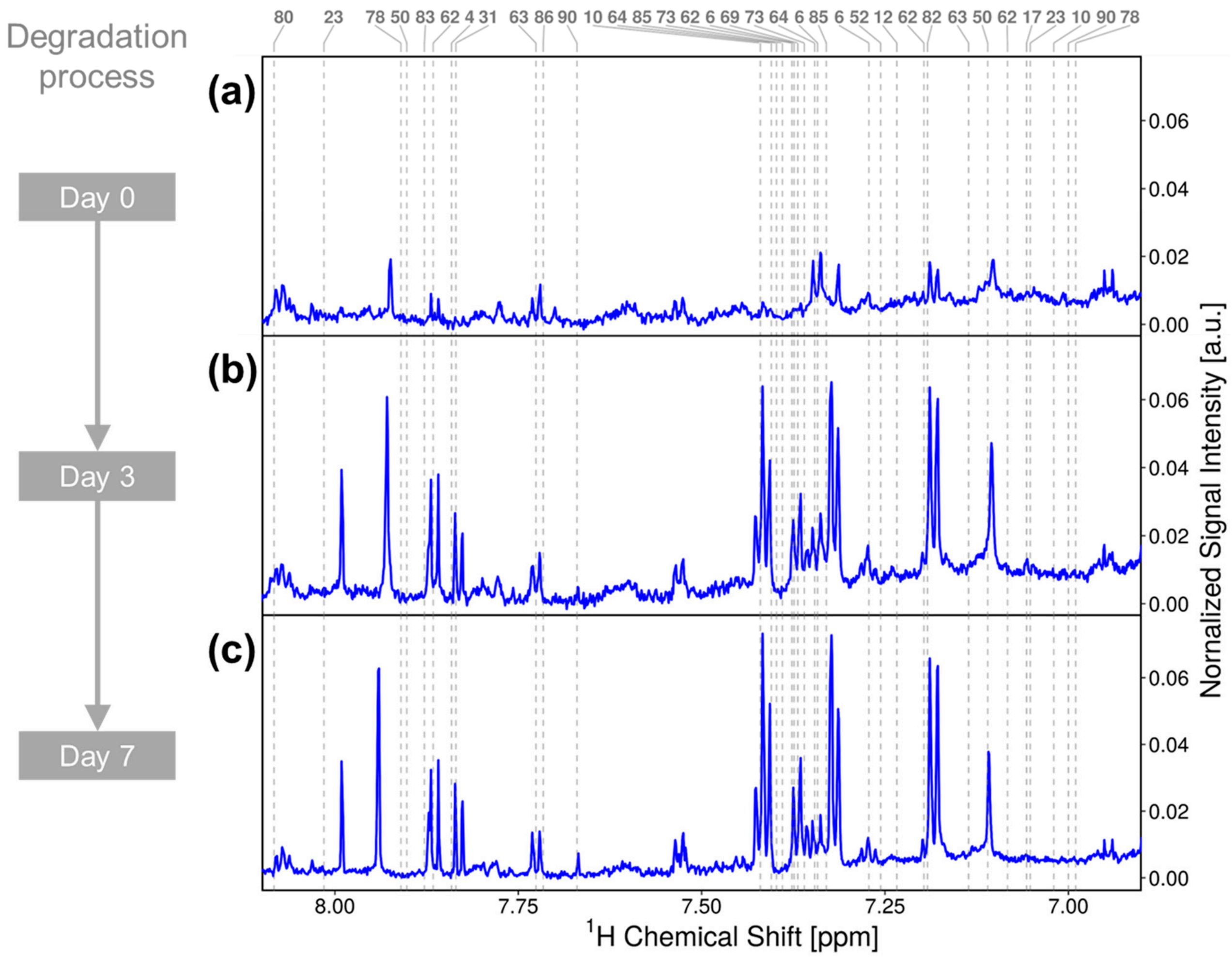

Figure S8. Continued.

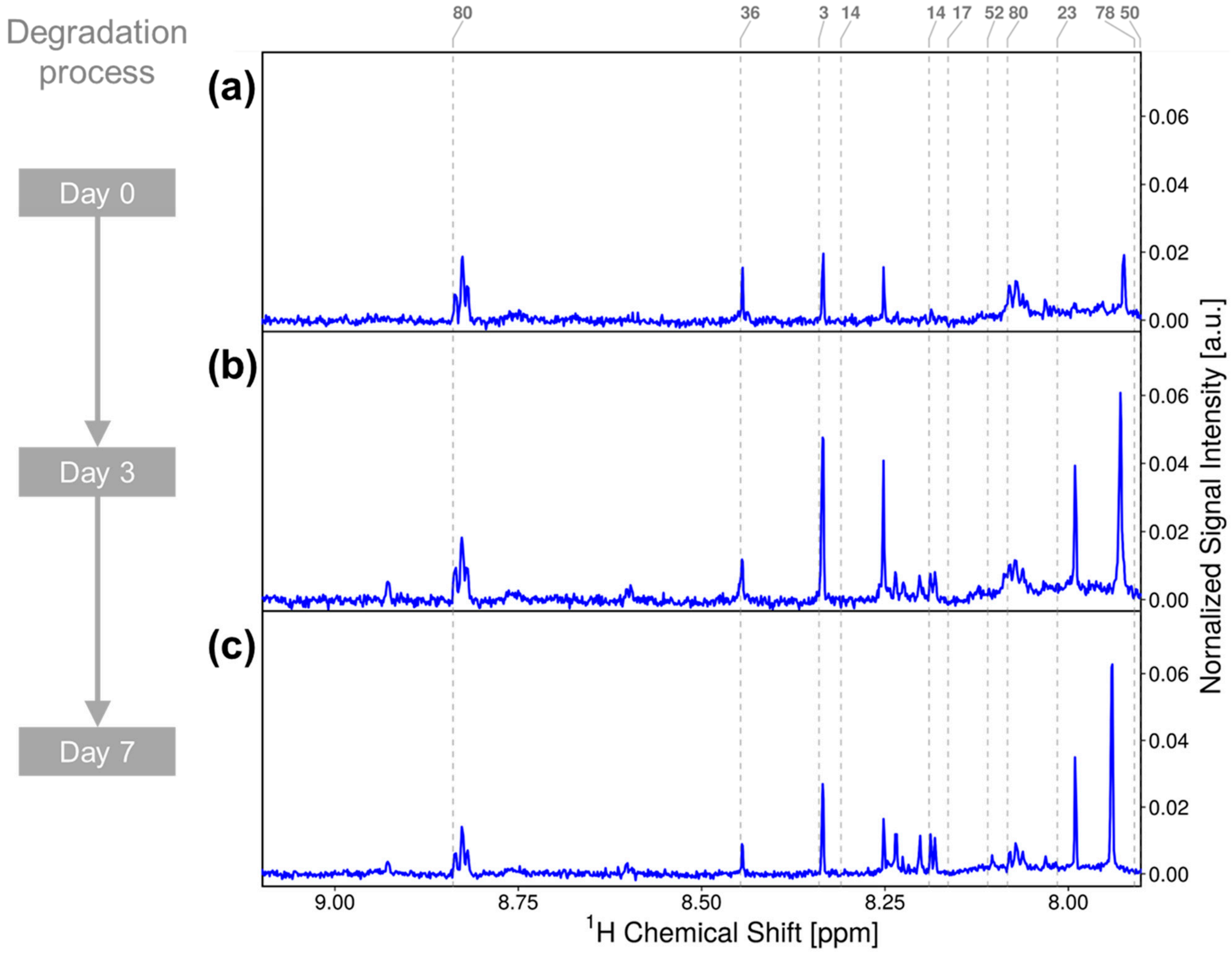

Figure S8. Continued.

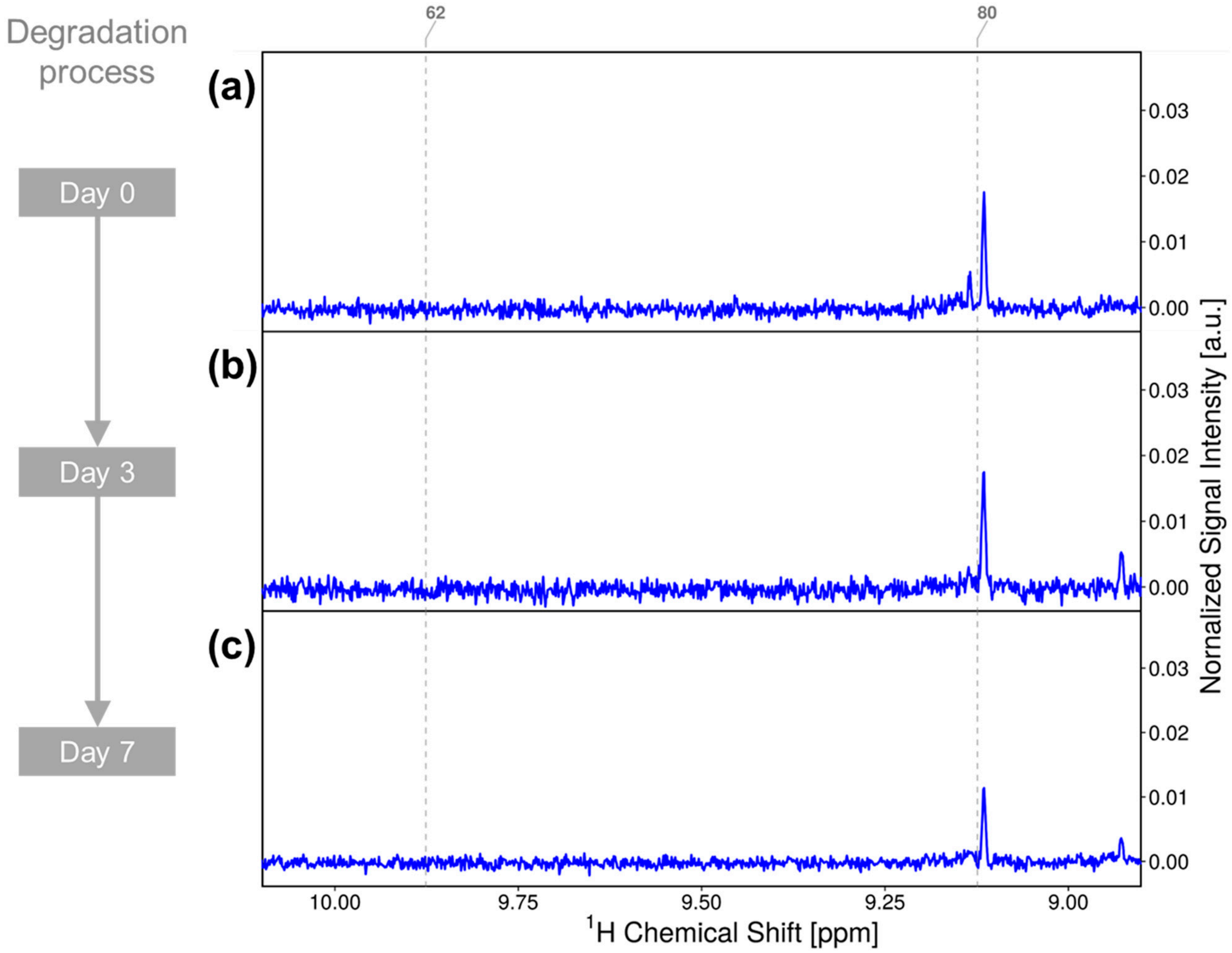

Figure S8. Continued.

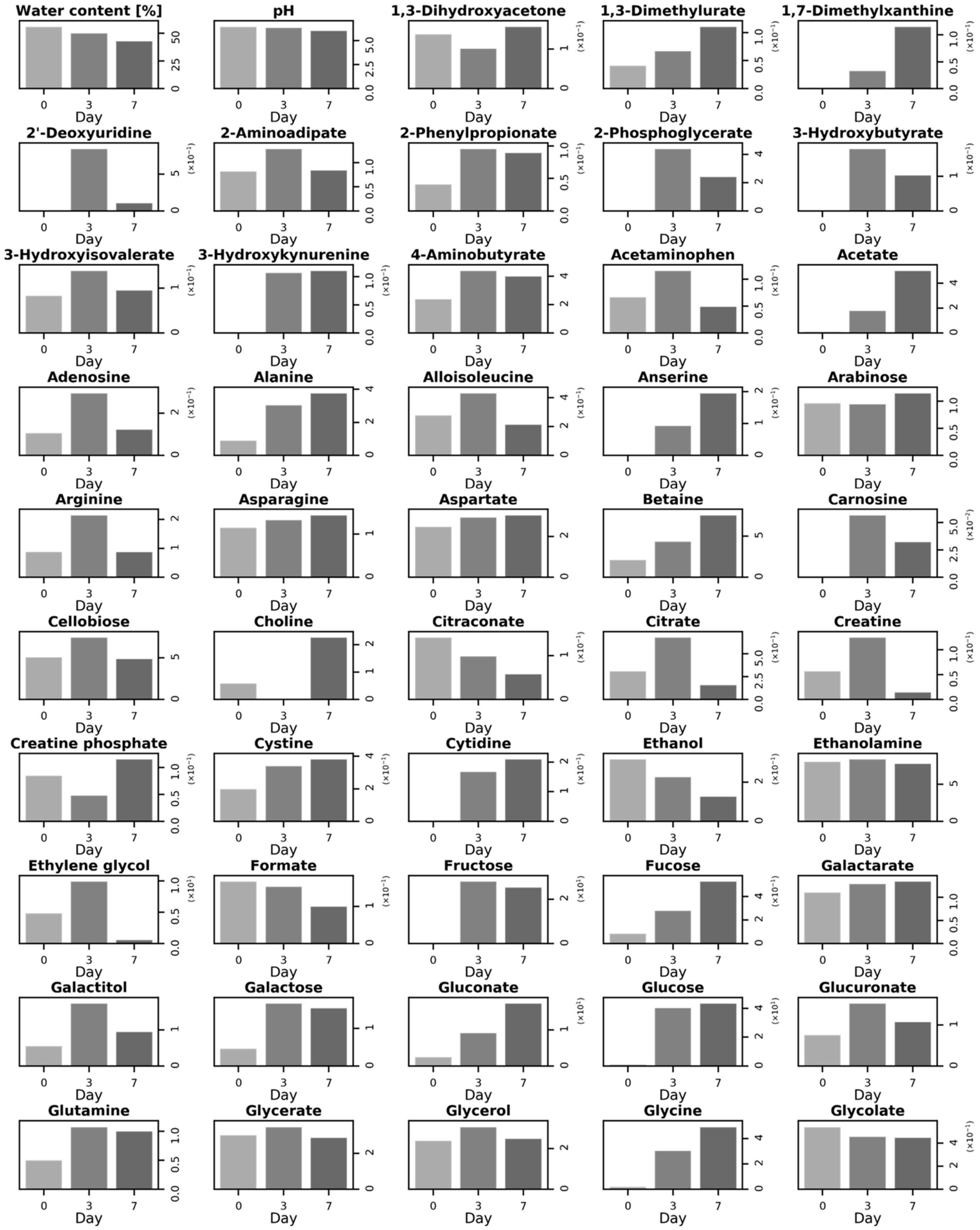

Figure S9. The transition of water content and pH in rice bran, and the amount of each metabolite in rice bran on each day of the control experiment. The amount of each metabolite was estimated with the Chenomx NMR Suite software using water presaturated 1D ^{1}H NMR spectra shown in Figure S8, and expressed as a relative ratio in comparison to DSS.

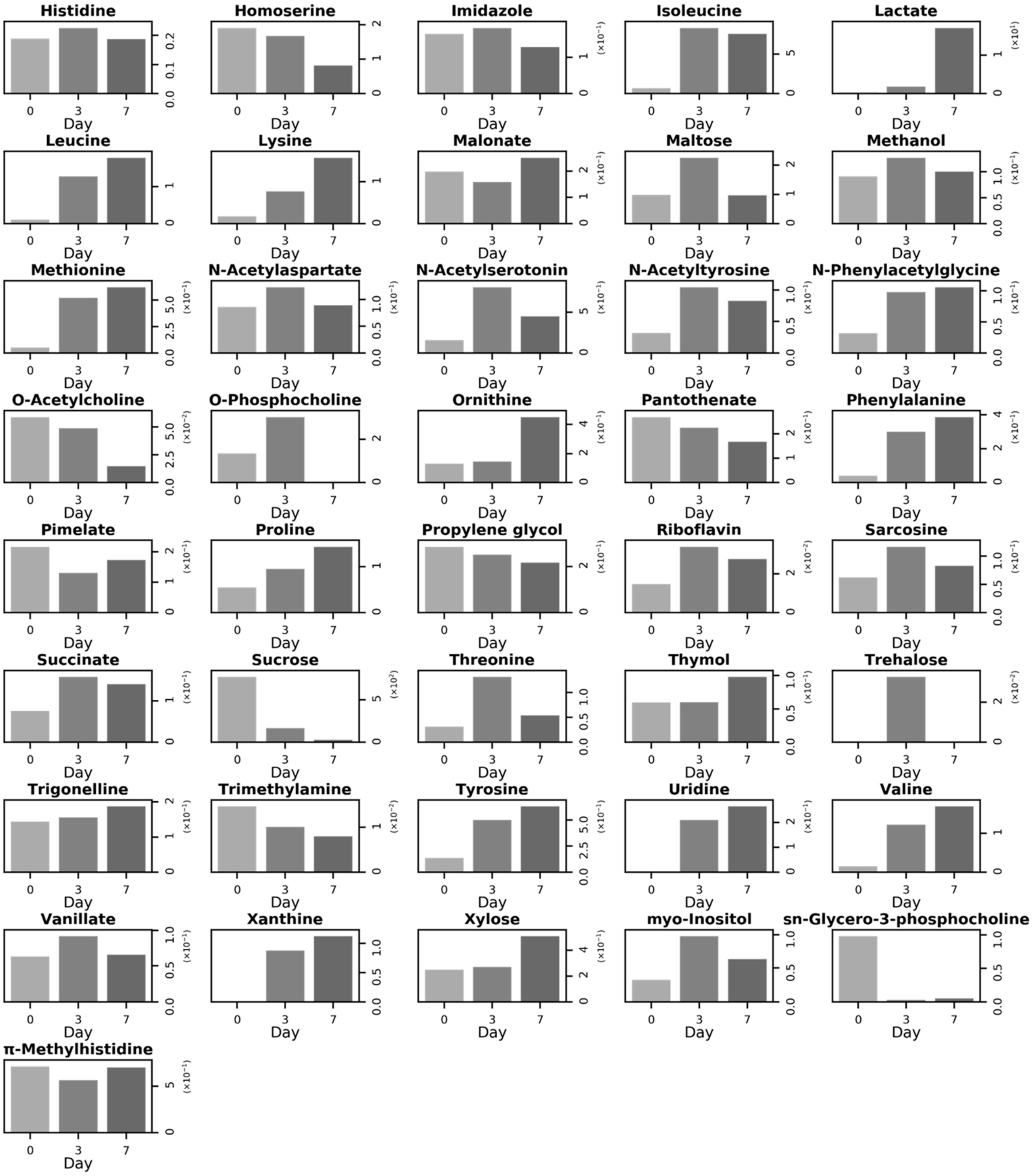

Figure S9. Continued.
